# Inclusive and Accurate Clinical Diagnostics Using Intelligent Computation and Smartphone Imaging

**DOI:** 10.1021/acssensors.4c01588

**Published:** 2024-10-15

**Authors:** Jisen Chen, Dajun Zhao, Hai-Wei Shi, Qiaolian Duan, Pawel Jajesniak, Yunxin Li, Wei Shen, Jinghui Zhang, Julien Reboud, Jonathan M. Cooper, Sheng Tang

**Affiliations:** † School of Environmental and Chemical Engineering, Jiangsu University of Science and Technology, Zhenjiang, Jiangsu 212003, PR China; ‡ Department of Cardiac Surgery, Zhongshan Hospital, Fudan University, Shanghai 200018, PR China; § Jiangsu Institute for Food and Drug Control, Nanjing, Jiangsu 210019, PR China; ∥ NMPA Key Laboratory for Impurity Profile of Chemical Drugs, Nanjing, Jiangsu 210019, PR China; ⊥ School of Pharmacy, Nanjing University of Chinese Medicine, Nanjing, Jiangsu 210046, PR China; # School of Engineering, University of Glasgow, Glasgow G12 8QQ, United Kingdom; ∇ College of Chemistry, Chemical Engineering and Materials Science, Soochow University, Suzhou 215123, China

**Keywords:** smartphone imaging, diagnosis, artificial intelligence, cardiovascular disease, skin tone, oximetry

## Abstract

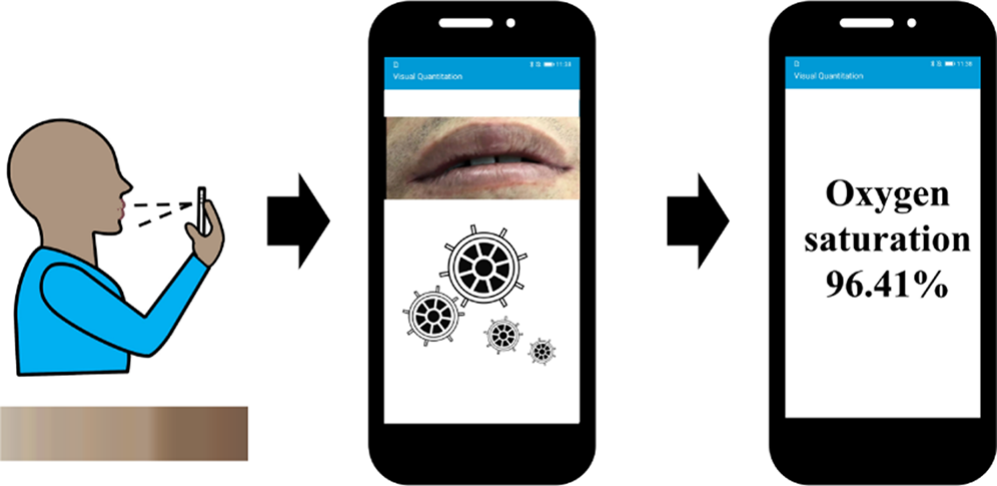

Smartphone-based colorimetry has been widely applied in clinical analysis, although significant challenges remain in its practical implementation, including the need to consider biases introduced by the ambient imaging environment, which limit its potential within a clinical decision pathway. In addition, most commercial devices demonstrate variability introduced by manufacturer-to-manufacturer differences. Here, we undertake a systematic characterization of the potential imaging interferences that lead to this limited performance in conventional smartphones and, in doing so, provide a comprehensive new understanding of smartphone color imaging. Through derivation of a strongly correlated parameter for sample quantification, we enable real-time imaging, which for the first time, takes the first steps to turning the mobile phone camera into an analytical instrument – irrespective of model, software, and the operating systems used. We demonstrate clinical applicability through the imaging of patients’ skin, enabling rapid and convenient diagnosis of cyanosis and measurement of local oxygen concentration to a level that unlocks clinical decision-making for monitoring cardiovascular disease and anemia. Importantly, we show that our solution also accounts for the differences in individuals’ skin tones as measured across the Fitzpatrick scale, overcoming potential clinically significant errors in current optical oximetry.

Following the design of digitalized color models by the International Commission on Illumination based upon human vision, the development of optical analytical systems has undergone significant advances,^1−3^ particularly through the potential use of smartphone-based imaging technology to enable convenient, low cost, and easy-to-use clinical^4^ and environmental sensors.^5−12^ During this period of development, the application of smartphone-based colorimetry (SBC) has however been constrained in its practical use by the influence of environmental conditions on the detection process.^1^ Images of the same individual, taken under different ambient lights often yield significantly different quantitative analytical results and thus generally require the use of external lighting or other auxiliary equipment.^9,11−20^ In clinical diagnostics, particularly in situations that require real-time analysis of images of the body (e.g., skin, lips, and fingernails), these inconsistencies lead to unacceptably high variations in analysis. For a RAW image, even the selection of the measurement area may cause a significant difference in the quantitative output.^21−24^ Of particular importance is the fact that the accuracy and reproducibility of the analysis are strongly influenced by camera settings, which are most often determined automatically,^12,13,15,23^ so limiting home use and constraining routine health monitoring.

A common solution to the ambient/environmental interferences is to shield the region of interest (ROI) from ambient light, most often either by using customized (e.g., 3D-printed) auxiliary equipment or by combining a separate external light source. For example, light-shielding cassettes with built-in LED light sources and sensitive signal reading devices (e.g., colorimetric multiarray readers) are most frequently used to ensure that the detection process is performed in an optimum environment.^11,16−20,25^ These modules lead to extra costs and limit the flexibility of operation, negating the universality of the smartphone as a simple diagnostic/imaging device.

Alternatively, the use of color calibration with the help of a standard or customized color card has been used,^19,22,26^ although the potential variability introduced from one manufacturer to another, coupled with differences in color mapping software in smartphones substantially affects analysis, thus limiting real-time analysis. Illuminance and difference in shooting distance under ambient light conditions also serve to illustrate the challenge of developing low-cost practical clinical diagnostics.

Beyond these physical implementations, Whitesides *et al.* introduced the mathematical processing of gamma correction, together with leveraging colorimetric absorbance to increase accuracy.^27^ Bakker and Soda further demonstrated that mathematical treatment of colorimetric absorbance can be used to overcome ambient light issues and enable the use of different devices (with different camera specifications).^24,28^

Here, we develop a strategy of high-precision, internal, algorithmic optical calibration that can be implemented without any auxiliary equipment, enabling fast and accurate clinical diagnosis using only a simple smartphone app (Figure 1). This new concept is underpinned by implementation of a strongly correlated quantitative parameter (SCQP, see Derivation of SCQPs for Colorimetric Quantification), with which the detection error is calculated by a smartphone app (giving accuracies comparable with those of a UV–vis spectrophotometer).

**Figure 1:**
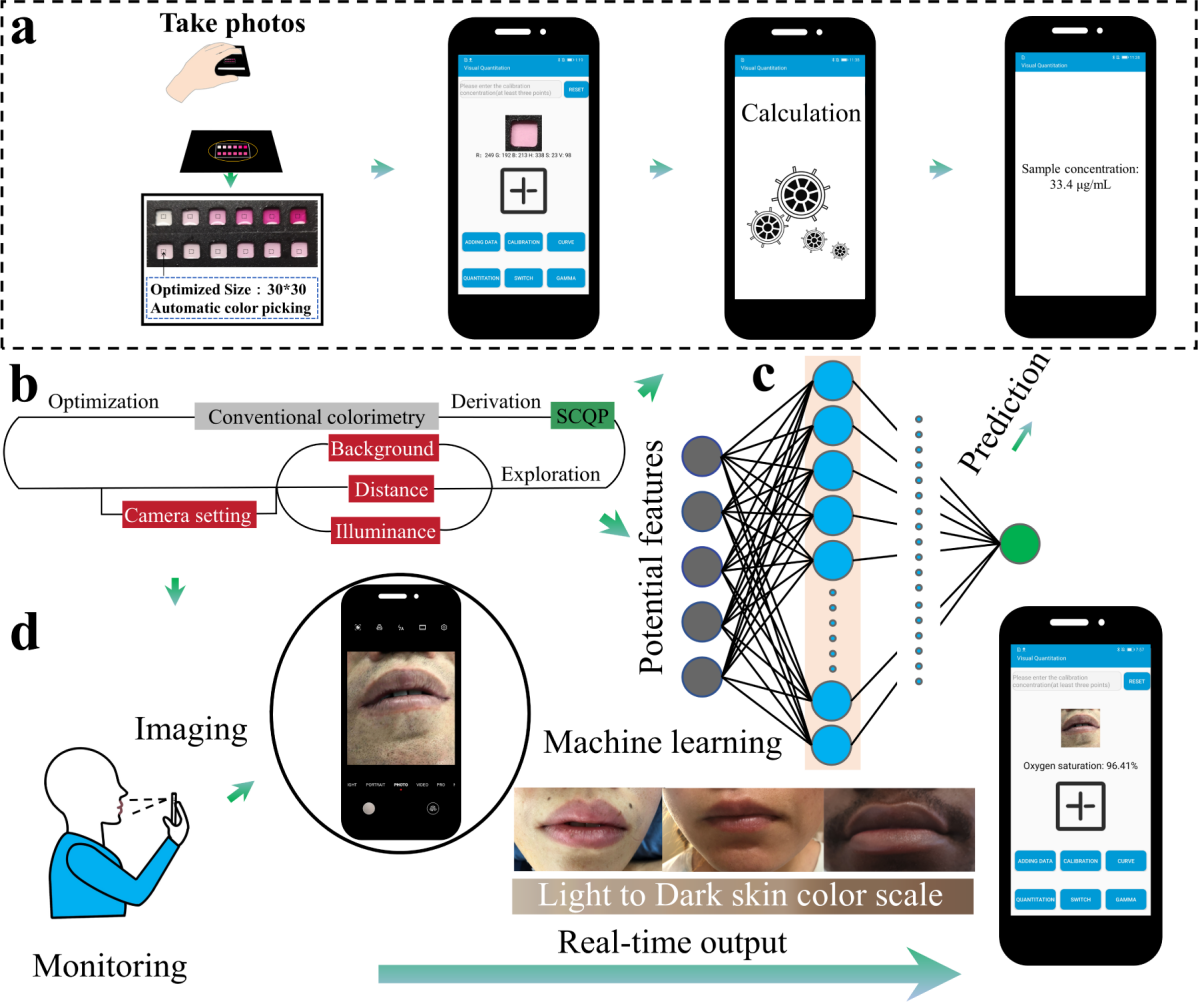
System overview. (a) Illustration of the smartphone app-based analysis, including sample imaging, signal extraction, fitting, and quantification, all completed in an app, with no expert intervention. (b) The optimization process compensating for (c) ambient interferences. (d) The application of the optimized system in clinical oxygen saturation monitoring in the lips (showing representative pictures with a range of skin tones), carried out by the app in a smartphone.

To achieve this, we systematically studied the environmental conditions that cause measurement error, including background, shooting distance, illuminance, and camera settings, to optimize the sensor’s performance. Through mathematical treatment as well as algorithm improvements, dominant interferences were successfully mitigated. To illustrate the application of this concept under resource-limited conditions (e.g., without standard samples), five machine learning models were validated, all showing excellent prediction accuracy using a trained model implemented in a smartphone app.

In order to demonstrate the success of our approach, a correction model was constructed to overcome challenges in pulse oximetry measurement, which we showed as being independent of important background influences,^29^ most notably the wide range of different skin tones in patients.^29−31^ We focused the application of our smartphone app to measure the oxygen saturation (SpO_2_)^29−31^ of patients with congenital heart disease (e.g., cyanotic heart disease) irrespective of both ambient condition, and, importantly, their skin tone.^29,32−34^

Patients with the pathological presentation of “cyanosis” (visualized as an aberrant blue coloring in human mucous membranes and skin^35^) are characterized by having an underlying disease where the deoxygenated hemoglobin level is more than or equal to 5.0 g dl^–1^. It is also an important indicator of hypoxemia (unusually low oxygenation levels (<80%) of arterial blood) and cyanotic congenital heart disease (CCHD).^32,33^ Areas of patients with a thin overlying dermis and rich superficial vasculature, such as the lips, finger or toe tips, earlobes, oral cavity, and extremities^34−37^ all provide potential positions where cyanosis can be screened, although currently conventional SBC methods are limited due to the interference of ambient light in home monitoring.

Traditional blood–gas analysis achieves high accuracy in SpO_2_ measurement and is used as a gold standard, but it requires specific and complex equipment, only available in centralized facilities, with invasive access to blood samples. While pulse oximetry is more convenient as it overcomes the invasive sampling through optical analysis, it is limited by high measurement errors induced by for example ambient light, perfusion level, and nail enamel, particularly between different skin tones,^29−31^ with significant clinical implications on challenges to establish an accurate diagnosis of hypoxemia.^29^

Using a smartphone-based imaging method has the potential to unlock accurate oximetry measurement,^29^ while measurement of partial pressure of CO_2_ (pCO_2_) can identify respiratory failures.^38^ For the latter, the current gold standard electrochemical methods have faced limitations due to the need for frequent calibration.^38^

Implementation of new methods for SBC enabled a detailed validation study to be performed, in which we demonstrate close agreement between smartphone analysis and arterial blood–gas analysis as a gold standard reference (see Table S7 for the comparison of different intelligent oximetry approaches). Image acquisition distance, illuminance, and camera settings were all mitigated, enabling oxygen saturation and pCO_2_ to be determined accurately from images of subjects’ lips, thus providing a precise low-cost point-of-care oxygen saturation diagnostic (±2.1%, mean |error| = 1.0%, RMSE (root-mean-square deviation) = 1.18%) with a sensitivity of 96% (95% CI (confidence interval), 79–99%) and pCO_2_ measurement (±5.9 mmHg, mean |error| = 2.74 mmHg, RMSE = 3.42 mmHg). This simple method for inclusive use can enable point-of-care or point-of-need home monitoring across a diversity of populations, with a step change in performance over existing systems, such as the finger pulse oximeter.

## Results

### Derivation of SCQPs for Colorimetric Quantification

Color spaces, such as RGB (red, green, and blue), HSV (hue, saturation, and value), or HSB (with B for brightness) more accurately describe the numerical relationship between wavelengths and human physiological response to observed color or color change in human vision. To first identify the most appropriate color space for our analysis, we performed a series of calibration experiments in which we first used emodin (red), which has a maximum absorption at 520 nm, as a model analyte imaged under ambient light, with a smartphone. Sample color in all spaces was provided by our app, validated by commercial software (including Adobe Photoshop and ImageJ^39^). The G (green) channel was applied as the quantitative parameter for linear fitting,^7,40,41^Figure S1a–c shows that measurements of different concentrations are significantly different from those obtained by UV–vis spectrophotometry, highlighting the challenges caused by external environmental interferences. The foremost contribution to this difference is ambient illumination, which may lead to diverse color stimuli.^21,23^

The color stimulus, also described as the spectral power distribution, is determined by the illumination and optical properties of an object (through its reflectance, adsorption, or transmittance) and represents the signal strength of the image captured by the camera. However, conventional RGB channels cannot quantify the change in illumination, requiring that HSV color space^1^ is introduced to describe the luminance component in our work (with *S* and *V* expressed as percentages).

To better understand this, we further explored the variations of each of *R*, *G*, *B*, *H*, *S*, and *V* against measurements with a standard reference method (UV–vis spectrometry, Figure S1a–c). The S value-based calculation had the smallest mean |error|, 2.46 μg mL^–1^, as also reported by Coleman et al.^42^ Importantly, the *S* value-based calculation has a limited linear range, and an obvious deviation would be apparent if the sample were at a low concentration (Figure S2a–d), resulting in a nonlinear *S* value (gray square in Figure S2). Thus, in order to test this hypothesis that deviations are related to the inherent definition of the S channel (see “SCQP” in Supplementary Methods, eq S1), and again, using red emodin as a model, we note that S can be approximated over the full range of concentrations by 1 – *G*/*V* (from 0 to 100 μg mL^–1^, Figure S2a, purple line, *R*^2^ = 0.995). Similar phenomena were also observed when copper sulfate (blue), ferric trichloride (yellow), and ink (green) are used as the model samples (Figure S2b–d). A robust grayscale picture can also be obtained from a weighted average of *R*, *G*, and *B* values, reporting an object’s brightness. This can lead to more accurate analysis than using the HSV color space (Figure S2), although *G*/*V*, derived from saturation, consistently outperforms. Our SCQPs (including e.g., *G*/*V*) demonstrated strong performance with the wide range of colors used in this study. However, the underpinning theory suggests a degree of nonlinearity with all color spaces as absorbance is logarithmically linked to light intensity. Consequently, the extrapolation of our results to other colors may require adapting parameters further.

### Background Interference

Camera settings (e.g., ISO, aperture, shutter speed) of a smartphone are often automatically adjusted according to the intensity of reflected light transmitted through the lens, with the possibility of variations of background color affecting measured color values (i.e., *R*, *G*, *B*, *H*, *S*, *V*), especially the *V* value.^21,23^ Using four different tones (black, red, green and white, Figures S3–S6), we explored the influence of different backgrounds on the quantitative results (with eight samples tested for each background, and the difference between the quantitative results of our system and that of the UV–vis detection shown in Figure S3). As expected, discrepancies in the *V* values from sample images taken in different backgrounds were observed (Table S1), with results indicating that the highest mean value of *V* (96.72%, ± 0.16%) was obtained in images with black backgrounds and the lowest with the white backgrounds (61.03%, ± 0.59%).

Bland–Altman analysis was used to evaluate the agreement between our app and UV–Vis detection (Figure S3),^43^ which was tested for normality (*p* < 0.05). The quantitative results of the image taken with the black background showed better consistency with the standard method (Figure S3a, bias = 0.04 μg mL^–1^) compared to the other three backgrounds (Figure S3b–d). Meanwhile, the 95% limits of agreement (LoA)^43^ included a lower level of overall quantitative discrepancy within ±4.45 μg mL^–1^ of UV–Vis data (Figure S3, 4.48 μg mL^–1^ and −4.41 μg mL^–1^).

The closeness between the quantitative results of the two methods is intuitively depicted with a regression line (Figure S3e–h). Furthermore, the app accuracy in black, red, green, and white backgrounds, obtained by calculating errors with UV–vis (mean |error| (black) = 1.84 μg mL^–1^, mean |error| (red) = 4.04 μg mL^–1^, mean |error| (green) = 4.92 μg mL^–1^, and mean |error| (white) = 2.30 μg mL^–1^), indicated that the analytical performance of our system in the black background achieved better stability and accuracy over the whole quantitative range of the sample. White balance-based correction is known to be more practical to stabilize the raw color of an image.^21^ We also explored the performance of our quantified strategy on a mixed background (Figure S5) and a background with different surfaces (Figure S6), dramatically reducing the error (compared to UV–vis ground truth) (below 3 μg mL^–1^ with our strategy to above 12 μg mL^–1^ without). However, the reflectance in different backgrounds is a crucial factor (and is, for example, an important influence for different skin tones in clinical measurement). To allow us to study other parameters (see Shooting Distance Interference and Calibration and Ambient Illumination Interference in Supporting Information for details) systematically, we used a black background for subsequent experiments (and ultimately included this parameter into our machine learning and clinical detection model).

### Detection Strategy with Machine Learning

Although the accuracy and precision of analysis were optimized, quantification is inconvenient as standard samples are needed. Thus, to further simplify the detection process and reduce the dependence on reference materials or standard color cards, a computation-enabled machine learning environment was developed for direct colorimetric detection under ambient light. Models including multiple linear regression (MLR),^44^ polynomial regression (PR), support vector machine regression (SVR),^45^ decision tree regression (DTR),^46^ and neural network (NN)^47^ were selected to construct the detection strategy. SCQP and the above-mentioned factors were all incorporated into these models as feature vectors to perform supervised learning (sample concentrations as labels), which also verified our study on influencing factors. Learning curves and prediction performance of these models are provided in Figure S7, with test samples including concentrations outside the labels.

The generalized performance of the final models was evaluated in 60 real-time imaging samples (Figure S7d,e,i–k), with the sample data continually added to the training set and with the errors of the model applied in the validation and training sets gradually converging and reaching a low coincident root-mean-square error (RMSE). The successful incorporation of this machine learning model greatly simplified the detection process and presented the possibility of practical clinical application.

### Skin Tone-Based Measuring and Correction

**Figure 2:**
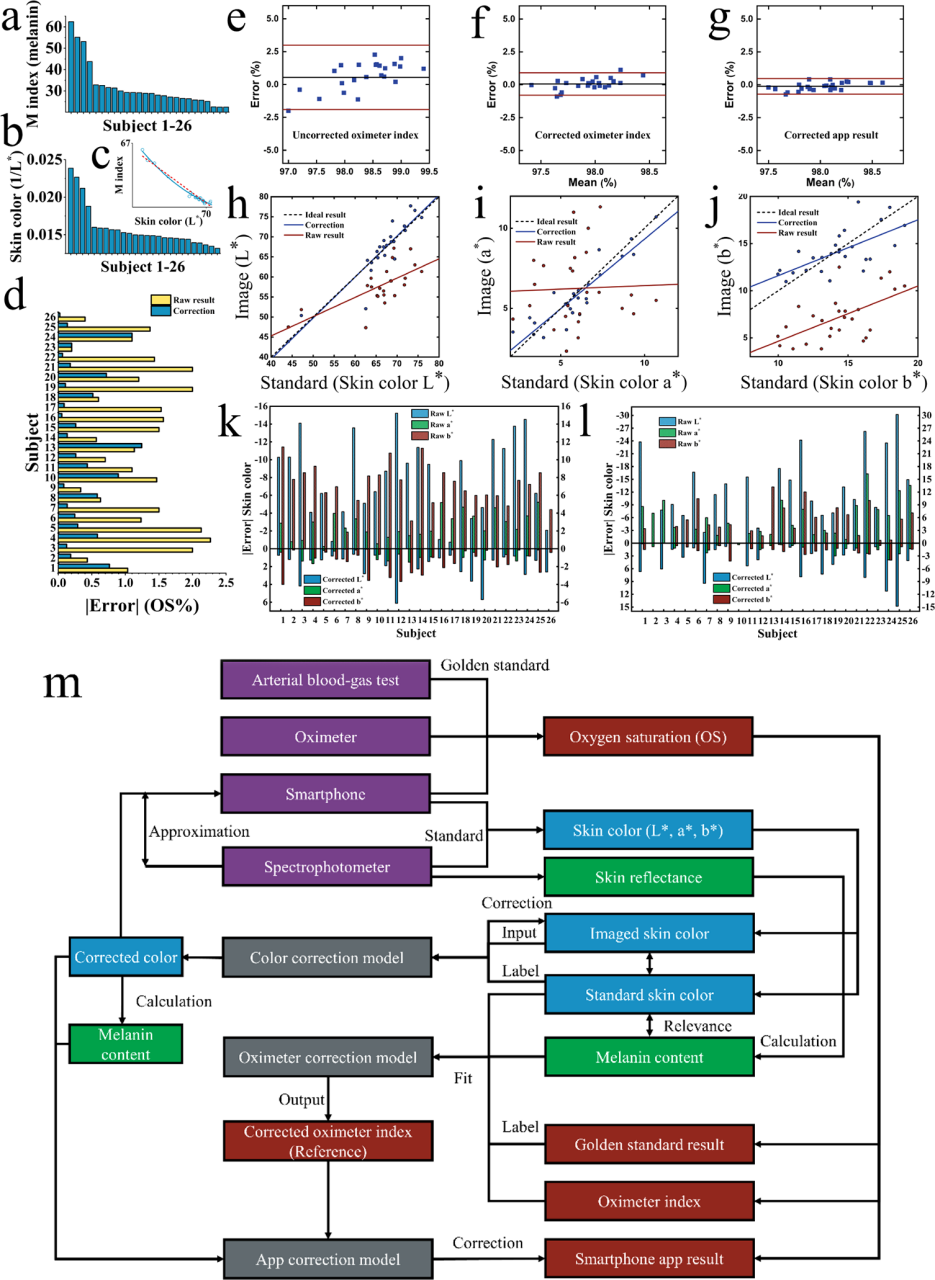
The interconnected correction system for oximeter and app accuracy. (a) Melanin content and (b) skin tone distribution of all the subjects in skin measurement (*x*-axis is subject number, with data provided in Table S2). (c) Correlation between melanin content and skin tone (L*): sigmoidal and linear fits. (d) Error distribution with subject number (yellow and blue bars are raw and corrected error between oximeter and golden standard, respectively). (e–f) Agreement between oximeter and gold standard in (e) raw result and (f) after normalization of skin tone-based correction. (g) Agreement between our corrected smartphone app method result and normalized oximeter index. (h–j) Comparison of corrected (blue solid line) and raw (red solid line) imaged skin tone (h-L*, i-a*, j-b*) with the ideal result (black dashed line). (k,l) Skin tone (L*, a*, b*) error between spectrophotometer measurement and imaged by a smartphone ((k) inner upper arm and (l) surrounding lip skin) before (up shadow bar) and after correction (down solid bar). The skin tone data details are given in detail in Tables S2–S4. (m) Workflow of the correction system. An oximeter correction model was used to fit standard skin tone, oximeter index, and melanin content, as features of the model, with the gold standard oxygen saturation result, used as a label for training (gray), and the corrected oximeter result was used as a reference (red), while a color correction model was fitted using standard (label) and imaged skin tone (gray). Our app correction model (gray square) was used to fit corrected skin tone and was compared with the corrected oximeter index (reference) to correct the error of app measurement, thereby eliminating the influence of different skin tones and obtaining improved accuracy. The correction strategy was investigated with an oximeter, arterial blood–gas test, and our smartphone app (purple square) to detect oxygen saturation and to produce oximeter index, see red square. Oximeter index, gold standard, and smartphone result. A spectrophotometer (purple) was applied to measure standard skin tone and reflectance and used to calculate melanin content (blue and green), with a smartphone used to take photos of skin to obtain imaged skin tone (blue).

Using our method, developed through the systematic analysis of image influencing factors, we established a normalization (see The Correction System for Oximeter Accuracy) to significantly increase the accuracy of oximetry measurements from simple images, for individuals with different skin tones (light to dark), using a common color space to index skin tone (Figure 2).^1,48^ (L*, a*, b*, as a device-independent color space determined in the CIE laboratory, in which L* indexes whiteness and blackness, while a* and b* represent the chrominance in red-green and blue-yellow, respectively.) The values of L*, a*, and b* are calculated from RGB values and can vary when using different cameras under nonstandard conditions.

We used the same systematic analysis as discussed above to understand and compensate for skin tone (L*, a*, b*), light reflectivity of skin, and melanin index (M index) measured by spectrophotometer, imaged color (L*, a*, b*, *R*, *G*, *B*, *H*, *S*, *V*) of skin and lip, camera setting (if supported, ISO), oximeter index, and the gold standard SpO_2_ result (from arterial blood gas measurements).

We present a skin tone scale and distribution of M index and skin tone parameter (1/L*) of all subjects using a spectrophotometer (Figures 1d and 2a,b). The M index has a sigmoidal relationship to light intensity (and thus to L). In our study (which covers a wide range of skin tones), this can be further approximated with a linear relationship (*R*^2^ = 0.97), Figure 2c.

The details of the corresponding Fitzpatrick,^49^ Von Luschan^50,51^ and Eumelanin Human Skin Color Scale^52^ (EHSCS) range of all subjects can be found in Table S2. Compared to the results of arterial blood–gas tests, those from different skin tone volunteers detected by a pulse oximeter confirmed the higher errors of the devices (Figure 2d, yellow bar; Figure 2e, mean |error| = 1.20%, low agreement with the gold standard). By modeling the relationship between content of melanin, skin tone, oximeter index, and gold standard result, we achieved a significant reduction in error (Figure 2d, blue bar; Figure 2f, mean |error| = 0.32%), recovering the benefits from an ambulatory measurement, with errors that would not lead to potential mis-treatment/mis-diagnosis. Similar results were obtained with direct imaging (Figure 2h–l), where poor results before correction were greatly improved using our smartphone method (Figure 2g, mean |error| = 0.26%), in real-time.

**Figure 3:**
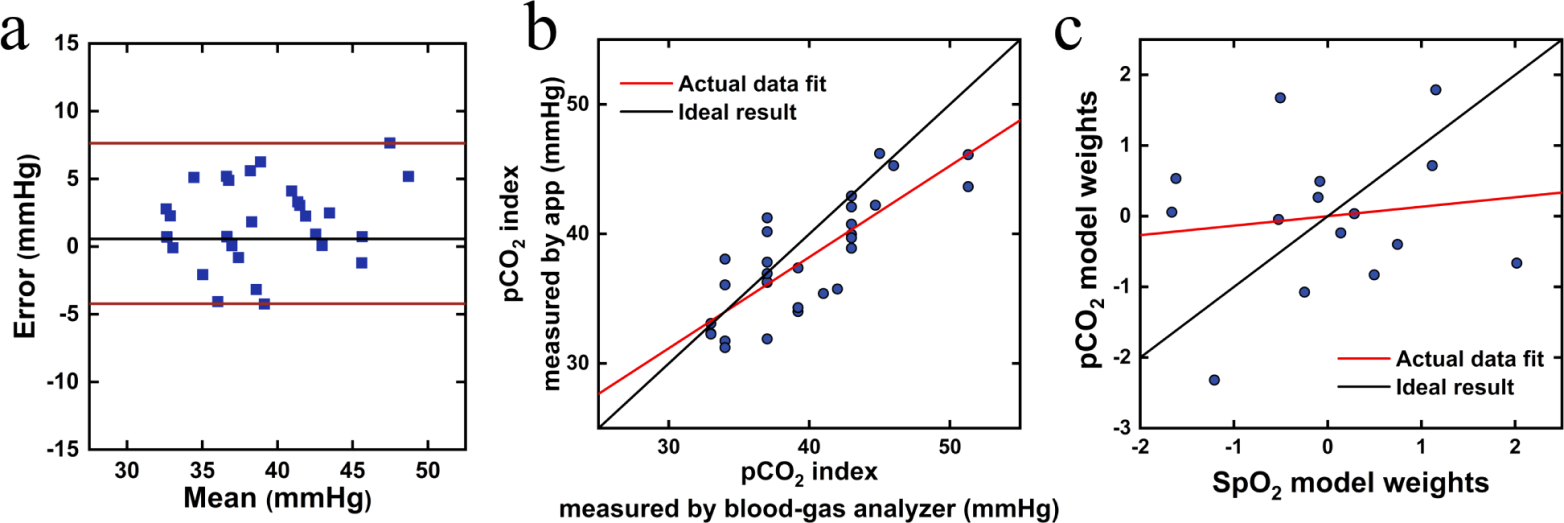
pCO_2_ detection. (a) Bland–Altman analysis showing the degree of agreement between our method and the gold-standard blood–gas analysis. The red and black lines represent 95% LoA (±5.9 mmHg) and bias (1.7 mmHg), respectively. (b) Regression analysis. Actual (red line) and ideal correlation (black line) reflect the pCO_2_ measurement performance of our system. (c) Regression analysis of SpO_2_ (*x*) and pCO_2_ (*y*) measurement models using both aligned weights of Z-score normalization. The result (*r* = 0.1) shows that there is no correlation relationship between them.

We further conducted a corresponding pCO_2_ index evaluation over the 26 subjects (see the Materials and Methods). The results represent the degree of agreement between our method and the gold-standard (blood gas analysis) (Figure 3a,b, mean |error| = 2.74 mmHg, RMSE = 3.42 mmHg, 95% LOA: ± 5.9 mmHg), within the published range (±7.5 mmHg).^38^ Both measurements of SpO_2_ and pCO_2_ were not obviously correlated (Figure 3c).

### Clinical Applications

**Figure 4:**
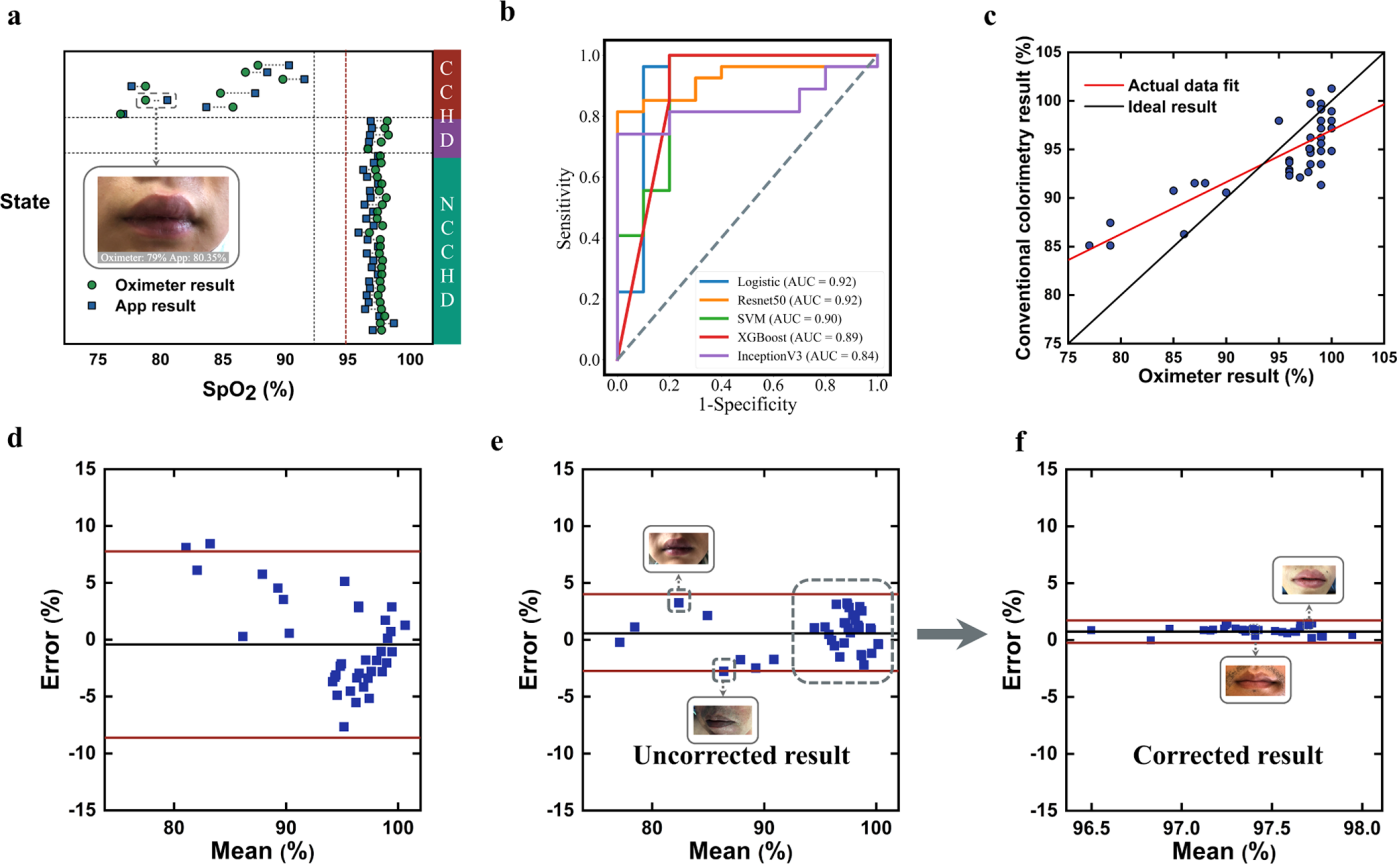
Clinical evaluation. (a) Clinical data matched with results obtained using an oximeter and developed app in different states of subjects after correction (red, CCHD subjects in a calm state; purple, the state of CCHD subjects after the auxiliary oxygen supply; green, NCCHD subject, mean |error| (mean absolute error) = 1.01%, RMSE = 1.18%. mean |error| = 1.57% before correction). The dashed red line represents the lower boundary of normal SpO_2_, and dashed black lines represent boundaries to differentiate state or type of subjects (see Tables S5 and S6 for performance comparison with gold standard blood gas analysis). Our corrective approach increases the accuracy of both devices, used at the “point-of-care”, against the gold standard. (b) ROC curves of 5 machine learning models. (c) Comparison of SpO_2_ results measured by the conventional colorimetric method (without considering interference of background illumination and skin tone, etc.) and oximeter. (d) Low agreement of the SpO_2_ detection results obtained by conventional colorimetric method (without considering interference factors such as background illumination and skin tone, etc., mean |error| = 3.53%, 95% LoA: 7.76% and −8.61%, bias = −0.42%) with oximeter, which present larger error than results of our app method that included interference factors (e). (e,f) Agreement of the detection results obtained by our app analysis before correction (e) and after correction (f) with oximeter index (e, the corrected results of patient samples in the dashed box are showed as f).

We then tested our smartphone app in measuring the oxygen saturation of patients with congenital heart disease (e.g., cyanosis and cyanotic heart disease) with different skin tones.^32−34^ As previously mentioned, standard measurements (laboratory-based oximeters and pulse oximeters) are either impractical for use in community or home settings or are prone to large errors. Areas with a thin overlying dermis and rich vasculature^34−37^ provide candidate sites where cyanosis can be screened, but conventional SBC methods have previously been limited by the interference of ambient light in home monitoring (Figure 4).

In our validation studies, a total of 26 subjects (Type II–VI on the Fitzpatrick scale) were selected, including 8 CCHD and 18 noncyanotic congenital heart disease (NCCHD) subjects. A total of 97 samples were collected in different environmental and physiological states. The lips of CCHD patients frequently presented an abnormal color in the calm state, and SpO_2_ was lower compared to that of NCCHD individuals. After auxiliary oxygen supplementation, the SpO_2_ and lip color of CCHD patients recovered (image set presented in Figure S8).

To establish an accurate and generic clinical model, the samples of 26 subjects were randomly split into a training set (58 samples) and a test set (39 samples) to minimize the influence of sampling bias. Before the training, the data set was standardized. The optimized clinical model was determined by error and performance analysis.

The performance of the clinical model is illustrated in Figure 4 and compared with the oximeter result, indicating that our method is comparable to SpO_2_ monitoring with a significantly lower error and conveniently using only a smartphone without extra equipment. The app results (with correction) of NCCHD patients agreed with the oximeter, while uncorrected results and conventional colorimetry did not. (Figure 4a,d, (a) points to the right of the red line, mean |error| = 1.0%.) We anticipate that a wider use of the app in clinical settings in the future will enable us to increase the strength of the models, consequently further improving the accuracy of the measurements.

It is important to note that the uncorrected oximeter results may be likely to lead to misdiagnosis or different and inappropriate treatment dosages, due to errors in underestimated SpO_2_ (Figure 4e, e.g., result_oximeter_ = 85%, result_gold-standard_ = 88.1%), while our real-time smartphone app measurements were very close to the gold standard and would not lead to a different interpretation of SpO_2_ (result_app_ = 87.8%). The use of the uncorrected oximeter could also lead to overestimation (Figure 4e, upside solid square, result_oximeter_ = 84%, result_gold-standard_ = 79%, and result_app_ = 80.8%), which can be life-threatening if the therapy was not given on time.

Moreover, combined with our strategy, we developed and tested 5 machine learning models to diagnose hypoxemia with a cutoff (≤95% as low SpO_2_ level).^53^ The receiver-operating characteristic (ROC) analyses of these models indicate that our strategy combined with machine learning successfully achieved good diagnosis performance with sensitivities and accuracies of 0.96 (95% CI, 79–99%) and 0.95 (logistic regression),^47^ 0.96 (95% CI, 79–99%) and 0.84 (Resnet50),^54^ 0.96 (95% CI, 79–99%) and 0.92 (SVM),^45^ 0.96 (95% CI, 79–99%) and 0.92 (XgBoost),^55^ and 0.74 (95% CI, 53–88%) and 0.78 (InceptionV3)^56^ (Figure 4b area under curve: 0.84–0.92).

Notably, in these models, we leveraged two deep learning models (Resnet50 and InceptionV3) to directly learn representations from the lip images, which also achieved good diagnosis capabilities. A T-SNE (t-distributed Stochastic Neighbor Embedding) clustering of all patient samples based upon clinical guidance around thresholds for SpO_2_ (Figure S9, red point, SpO_2_ < 95%, green point, 95% ≤ SpO_2_ < 97%, blue point, 97% ≤ SpO_2_ ≤ 100%),^53^ confirmed the ability of our app in monitoring and early warning of disease. The red points (hypoxemia) were automatically separated at an area (the left lower position). The green points that may need to give notice in hypoxemia were at the right lower position. These results indicate that the clinical features that we explored are promising diagnostic indicators for informing clinical care pathways.

## Discussion

This systematic study of the wide range of ambient and environmental parameters involved in colorimetry measurement accuracy has enabled us to build a robust correction model and introduce a machine-learning-based detection system into a smartphone diagnostic. We anticipate that the performance of the model will increase with more samples being used for training, as highlighted in Figure S24, although the existing model already brings significant improvements. This new understanding could in the future be applied to a wide range of imaging applications, including those involving diagnostic imaging of the eyes (in detecting retinopathies) or dermatological conditions, in decentralized settings in communities.

We demonstrated the applicability of this new smartphone-based method (app) for simple and noninvasive measurement of oxygen saturation by analyzing lip images in real-time, without any auxiliary device assistance, enabling remote diagnosis, clinical decisions, and disease screening (e.g., hypoxemia). We focused on lip images to create a method that does not require any additional equipment.

Although fingers have potentially fewer constraints to establish strong privacy-preserving confidence in patients, their color is not sufficient to enable accurate SpO_2_ measurement. In current medical practice, pulse oximeters can be placed on fingers, although recently the failings of these methods for people of different skin tones have been well documented. Currently, such systems now require specific equipment and lighting arrangements, limiting their applicability. In this context, Tables S5 and S6 demonstrate that our app provides results with an error below 1.3%, for all skin tones, when compared to that for a conventional blood gas analyzer (with a value that is below the published CV 2%)^29^ of the equipment used in this work (and clinically).

All error calculations are in accordance with the US FDA regulations for pulse oximetry.^57^ In 16 patients (considered in Table S5), the measurements with pulse oximetry had large enough errors that the patients would have had a high chance of being misdiagnosed. This relates to the recorded difficulties that the devices have in certain situations,^30,31^ where their errors do not meet the threshold ≤3%, set by International Organisation for Standardisation (ISO)-standards and required for Food and Drug Administration (FDA) 501(k)-clearance. Such errors can lead to “occult” hypoxemia, where arterial blood oxygen saturation is <88% despite a pulse oximetry reading >92%.^30,31^ Although the pulse oximeters fail in 3 out of 4 skin tone categories, our app measurements are well below the regulatory threshold and would avoid any instance of occult hypoxemia.

Contrary to other approaches in developing smartphone-based imaging techniques, our work uniquely focused on building the underpinning understanding of environmental constraints limiting previous devices. In other research, the methods overcame technical limitations by restricting applicability (e.g., to specific skin tones^58−60^ or using ancillary devices for calibration^59,60^). Our approach enables real-time measurement without any external devices or consideration of the environment (such as lighting). The understanding built within the method has the potential to unlock the application of smartphone imaging in a wide range of new applications.

## Materials and Methods

### Oxygen Saturation Measurement

Oxygen saturation was calculated by detecting the percentage of oxygen-bound hemoglobin (i.e., blood oxyhemoglobin),^37^ and that of three forms depicted as arterial (SaO_2_), venous (SvO_2_), and percutaneous oxygen saturation (SpO_2_), often measured with an oximeter.^34^ We performed measurements using 3 methods: (i) blood gas analysis using conventional, gold standard techniques, involving phlebotomy, (ii) pulse oximetry [Yuwell, YX306], and (iii) our newly developed smartphone imaging app.

### The Smartphone App

Univariate and multiple linear regression, region of interest (ROI) selection, edge threshold segmentation (see Optimization of the Edge Segmentation Algorithm and Signal Extraction in Supplementary Methods), brightness equalization correction (see Image Evenness Calibration Algorithm), color (three color spaces and their conversion including RGB, HSV, and L*a*b* were supported in our app) extraction, customized interference filtering were integrated into the app to optimize processing factors that interfere with the detection results in conventional smartphone colorimetric analysis. The workflow of the whole system is illustrated in Figure 1a–d. A plastic chip using photosensitive paper as the base material allowed sample loading into 6 rows with each containing 6 square grooves with 5 mm sides and 2 mm depth. The real-time photos of the chip with the samples (including standard samples and test samples) were taken using the app, or sample photos that were previously taken can be imported from the mobile phone album. Two algorithms were developed to extract the signal and filter shadows or light reflection spots. To reduce the error generated from the illumination, a multiscale retinex with color restoration (MSRCR) algorithm^61^ was applied for image processing.

### Skin Reflectance and Color Measurement

Subjects were recruited from across the Fitzpatrick scale for skin tone (Type II–VI) following the guidance from the US Food and Drug Administration of testing oximeter accuracy (Figure S8 – skin tones from light to dark referenced on Dadzie et al.^52^) without a pigmented lesion. According to the reported method,^52,62−64^ skin tone and reflectance of each subject were measured by a portable spectrophotometer (Spectrophotometer (CS-410), Hangzhou CHNSpec Technology CO., Ltd., China), Figure S10. The relationship between the melanin content (*M* index) and reflectance (655 nm) is shown below:
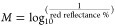
1

Four body parts (left and right upper inner and outer arms) were measured to obtain M index and skin tone three times (Figure S10). The averages of inner arm data over the two arms were used to approximate a standard baseline skin tone.

### The Correction System for Oximeter Accuracy

The oximetry error owing to different tones was corrected by an oximeter index, standard skin tone, and melanin content (eq 2). The standard skin tone, melanin, and imaged skin tone taken by the smartphone were modeled (eqs 3–6), to enable correction without specialized instruments. We performed standard skin measurements and skin imaging on each subject before SpO_2_ monitoring. Then, arteria radialis puncture was performed to obtain a blood sample for arterial blood–gas tests [RADIOMETER, ABL9], and at the same time, an oximeter [Yuwell, YX306] was used to continuously monitor SpO_2_ index, while photos of the individuals’ lips were taken by smartphone. Based upon these results, we constructed the correction system as follows:
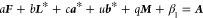
2
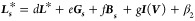
3
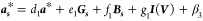
4
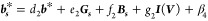
5
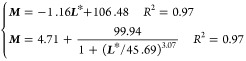
6

where ***M*** is the skin tone measured by a spectrophotometer – L*, a*, b* and oximeter index – ***F*** and SpO_2_ result of the arterial blood–gas test – ***A***. Skin tone imaged by a smartphone – *L*_s_*, *a*_s_*, *b*_s_*, *G*_s_*, *B*_s_*, L*, a*, b*, ***M*** and camera setting – ISO (***I***, if it cannot be supported by some smartphones, it can be replaced with brightness – ***V*** in HSV color space). β_*i*_ are constants, and scalar terms are weights of the correction algorithm.

### Image Evenness Calibration Algorithm

Based on the MSRCR algorithm,^61^ a simple and lightweight image evenness calibration algorithm was developed for smartphones. Specifically, to keep the overall image undistorted, only the *V* value was optimized, and multiscale Gaussian filters^61^ were applied to denoise the sample image and highlight areas with strong features. Then, the app algorithm was used to perform deviation equalization on the extracted *V* matrix (here, *V* values ranged from 0 to 1) from the sample image. After re-encoding based on the equalized color value of each pixel, quantitative analysis was completed through the new output image (Figure S22) as follows:
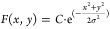
7
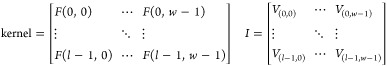
8
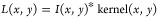
9
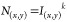
10

Here, a two-dimensional Gaussian function was selected as *F*(*x*,*y*). *C* is a normalized constant, where *C =* 1/(∑*x*∑*yF*(*x,y*)). In this work, for dynamic range compression and image feature detail, three scales (σ = 20,72,250) were selected. *I* and *L* represent the V value matrix from the raw image and postconvolution image, respectively; kernel represents the Gaussian convolution kernel, which presents the calculated result of every pixel (*x*,*y*) by *F*(*x*, *y*), and * is the convolution operator. *N* is the V result matrix after calibration, and *k* is the calibration index.
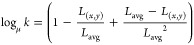
11

Eq 11 was used to complete the deviation equalization. It presents the relationship between *k* and μ, and μ ranges from 0 to 1. A smaller μ leads to a greater degree of calibration (Figure S23). *L*_avg_ represents the average of the *L* matrix values.

### Shooting Distance Interference Calibration

The formula fitting-based calibration strategy was as follows:
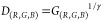
12
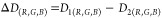
13
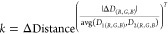
14
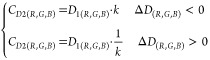
15
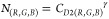
16
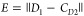
17where *G*_(R,G,B)_ is the color value of the output image on the screen after a conventional GAMMA correction of *D*_(R,G,B),_ and *D*_(R,G,B)_ is the ideal color value of the input image detected by the sensing device (e.g., CCD, CMOS) of the camera.^22,24^ τ and ΔDistance are the amendment factor and difference in distance between the two images, respectively. 1/γ represents the inverse correction index.

In our experiments, ΔDistance = 5 and γ = 2.2 (γ is a GAMMA correction factor,^24^ note that it depends upon the internal correction and ranged from 2.0 to 2.2). To eliminate the error between the sample signal obtained from different distances and achieve normalization, a Δ*D*_(R,G,B)_-based exponential function of the distance was introduced. *D*_1(R,G,B)_ and *D*_2(R,G,B)_ are *D*_(R,G,B)_ of samples at different distances. *C*_D2(R,G,B)_ is the calibrated result of the *D*_(R,G,B)_ values. After elimination of the error caused by the distances, the *D*_(R,G,B)_ values were transferred to *C*_D2(R,G,B)._*N*_(R,G,B)_ was recovered as an output color value from the *C*_D2(R,G,B)_. τ was adjusted to be 1.1 for our algorithm (eq 17, error of the values for *D*_1_ and *C*_D2_). See “Shooting Distance Interference and Calibration” in the Supporting Information for details.

### Experienced Features and Regression Models

Regression models, including MLR, PR, SVR, and DTR, were introduced to the detection strategy based upon machine learning. L1 regularization (Lasso regression)^65^ was added to the MLR and PR models. Architectures and details of the DTR model are presented in Figure S11. The MLR, PR, and SVR detection models were designed based on the following:
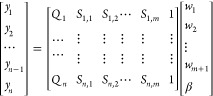
18
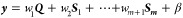
19

Here, ***Q***, ***S***_**1**_···***S***_***m***_, and ***y*** are vectors with *n* dimensions. ***Q*** is the SCQP (G/V) vector. *w*_1_···*w*_*m*+1_ are weights. β is a constant term. ***S***_**1**_···***S***_***m***_ are the different influencing factor vectors explored in this work (illuminance, shooting distance, and camera setting). These values were explored as experienced features in our experiments. ***y*** represents the result vector.

Specifically, square features were added to the PR model using the following formula:
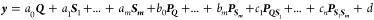
20

Here, *a*, *b*, and *c* are weights. ***P*** is the feature with polynomial combinations.

### Loss Function and Model Training

Training of the DTR model was based on a CART.^46^ The MLR and PR models in this study were trained by minimizing the following loss function (Lasso regression):
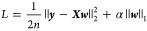
21where ***X*** is the feature matrix of the training sample, and ***w*** represents the weight matrix. Here, α = 0.1, which represents the degree of L1 regularization. The SVR model was trained by minimizing the following loss function:
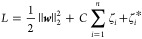
22
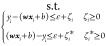
23where *y* and ***x*** are the concentration and feature vectors in the obtained data from real samples, respectively, and ζ and ζ* represent the relaxation factors of the upper and lower support bounds. *C* = 1.5, and ε = 1 in this model.

Unless otherwise indicated, the classification models were trained by minimizing the following loss function:
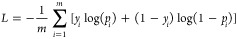
24where *y* is the true sample label, and *p* is the result of class probability of our sample predicted by model.

These models were optimized by stochastic gradient descent (SGD) using the Python package sklearn,^66^ and the maximum depth was set to 3 in the DTR model. K-fold cross-validation was applied to evaluate these models in training (here, *K* = 10).

### Subject pCO_2_ Measurement Model


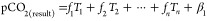
25where *f*_1_···*f_n_* are the model weights, and *T*_1_···*T*_*n*_ represent the cross-dimensional lip and skin tone parameters corresponding with pCO_2_ measurement obtained from the smartphone app. β_1_ is a bias term. We adopted noise-based training^67^ for learning the potential pattern of change in various environments, in which noise was randomly added into 20% of the training sample. Specifically, taking a feature value *t*_*m*_ of vector *T*_*m*_ as an example, feature *t*_*m*_ can be depicted as *t*_*m*_ = *n*_*m*_ + *o*_*m*_, if noise *n*_*m*_ was added in the training sample *m*. Otherwise, *t*_*m*_ is equal to *o*_*m*_. *o*_*m*_ is the raw value of the feature extracted by the app.

### Deep Learning Models

The first neural network with three layers was implemented using Keras.^68^ The first (input) layer had 5 neurons to load features. Four fully connected latent layers with rectified linear unit (ReLU) activations of 64, 32, 16, and 5 neurons followed the input layer, which was ultimately connected with an output layer of 1 neuron.

For hypoxemia diagnosis, we used transfer learning based on Resnet50^54^ and InceptionV3^56^ architecture and fine-tuning weights of models, which were implemented using Keras.^68^ We adopted an early stopping strategy in training, which was triggered by the no loss value decreasing over the validation data set within 15 epochs.

### Clinical Model

Inspired by a recent study regarding clinical hemoglobin/anemia analysis,^48^ the analytical model developed is as follows:
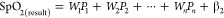
26where *W*_1_···*W*_*n*_ are the model weights, and *P*_1_···*P*_*n*_ represent the cross-dimensional lip and skin tone parameters corresponding with SpO_2_ measurement obtained by the smartphone app (details in Figure S12). Three color spaces were used in the analysis: RGB for identifying features (lip and skin), HSV for color-based oximetry, and Lab* for skin tone. These features were designed based on our systematic exploration to interference. β_2_ is a bias term of the model. The sample images are provided as a set in Figure S8. RMSE of SpO_2_ measured by our clinical model within subjects of different skin phototypes is presented in Tables S5 and S6. Training strategy of our clinical model was same as for the pCO_2_ analysis model (see Subject pCO2 Measurement Model).

### Clinical Research Overview

The clinical research was approved by the Ethics Committee of Zhongshan Hospital (approval number B2022-314R). All subjects (*n* = 47) agreed to and signed informed consent before participating in the study. The clinical experiments were completed under relevant ethical guidelines for human subject research (the Declaration of Helsinki), under the supervision of professional doctors. Before recruitment, our study group organized a presentation to describe the project and the benefits of taking part. Each candidate was informed of the study details in writing and agreed to take part through an informed consent document.

We recruited subjects that ranged from 15 to 80 years old. Subjects with other diseases that could influence the lip color were excluded. Inclusion criteria are as below:

Cyanotic congenital heart disease group: patients of pulse oxygen saturation of right finger or toe (SpO_2_) <90%, or the result of the interval >1 h for 3 consecutive monitoring SpO_2_ in 90–94%, or SpO_2_ difference between right finger and toe for 3 consecutive times in 1 h >3%, and cardiac ultrasound confirming congenital heart disease.

Noncyanotic congenital heart disease group: patients of SpO_2_ of right finger or toe <95%, and cardiac ultrasound confirming congenital heart disease.

After admission to the hospital, the right finger or toe SpO_2_ of each subject was collected three times by oximeter at intervals greater than 1 h, and lip images were collected at the same time for app analysis.

All statistical test results were obtained using Origin 2018 (OriginLabs, v2018C, SR1). Comparison of different intelligent oximetry approaches are presented in Table S7.

### App and Algorithm Development

All of the algorithms and models mentioned in this study were incorporated into mobile apps. The beta version of the app was developed with the open-source integrated development environment (IDE) Android Studio (Google, Mountain View, CA) and IntelliJ IDEA (JetBrains, Community Edition). The machine learning models were programmed by PyCharm (JetBrains, Community Edition) and transformed using TensorFlow^69^ Lite.

## Data Availability

The source code, including the algorithm and model, is available from Github repository [available at https://github.com/Jisencc/ICSO]. Any commercial use, including the distribution, sale, lease, license, or other transfer of the code to a third party, is prohibited. The deidentified clinical samples (lip images) and other underlying data are available from the University of Glasgow repository at http://dx.doi.org/10.5525/gla.researchdata.1784.

## Supporting Information Available

Supplementary Text: shooting distance interference and calibration, ambient illumination interference, robustness and compatibility; Supplementary Methods: detection processes, SCQP, optimization of the edge segmentation algorithm and signal extraction, and sample preparation (PDF)
Demonstration of the app measuring a patient (MP4)
Demonstration of the analysis of standards of different colors utilizing the app (MP4)
Demonstration of loading samples and measuring them using the app (MP4)

## List of abbreviations

SBC: smartphone-based colorimetry (SBC)
SCQP: strongly correlated quantitative parameter
SpO_2_: peripheral oxygen saturation
RMSE: root-mean-square deviation
CI: confidence interval
t-SNE: t-distributed stochastic neighbor embedding
