# Supplementary material for: Inclusive and Accurate Clinical Diagnostics Using Intelligent Computation and Smartphone Imaging
Source: ACS Sens. 2024 Oct 15;9(10):5342–53. doi: 10.1021/acssensors.4c01588 (PMC11519924; doi:10.1021/acssensors.4c01588)
Supplement: Supplementary file 1 — se4c01588_si_001.pdf [file se4c01588_si_001.pdf]

Supplementary Information for

Inclusive and Accurate Clinical Diagnostics using Intelligent Computation and Smartphone Imaging

Jisen Chen, Dajun Zhao, Hai-Wei Shi, Qiaolian Duan, Pawel Jajesniak, Yunxin Li, Wei Shen,* Jinghui Zhang, Julien Reboud, Jonathan M. Cooper,* and Sheng Tang*

Corresponding authors
Emails: Jon.Cooper@glasgow.ac.uk; tangsheng.nju@gmail.com; shenweivv@126.com

This PDF file includes:

Supplementary Text
Supplementary Methods
Figures S1 to S24
Tables S1 to S9
SI References

Other supporting materials for this manuscript include the following:

Movies S1 to S3

Supplementary Text

Shooting distance interference and calibration. When the shooting distance (from the sensor to the sample) changes, the proportion of the sample area in the entire image also changes, resulting in differences in the light reflection from the background. As shown in Fig. S13a, the relative error (RE, ranged from 0.01 to 0.31) increases, especially at distances > 20 cm, revealing that the shooting distance has a significant influence on the quantitative results (Fig. S13a, Pearson's r = 0.87). Using 330 sets of data, a formula fitting-based algorithm was implemented to correct the error (see "Shooting distance interference calibration" in Methods, Equation (12)-(17) and Fig. S13d-i). We also evaluated the formula in three subjects, imaged at four distances, demonstrating such optimization enables customized SpO_2 measurement to each user (to reduce the distance limit, Fig. S14).

Using this correction algorithm, an iterative calculation in our app was processed to minimize the quantitative error caused by changes in the shooting distance (Fig. S13, d-i). Furthermore, the optimal quantitative distance for each sample was obtained by iteratively invoking this algorithm. After this calibration, the original color values (Fig. S13, d-i, dashed red line) of each distance were converted (Fig. S13, d-i, dashed blue line). The algorithm was applied to data measured by a smartphone with automatic camera settings, providing a method to correct the strict bias (Fig. S15).

Ambient illumination interference. As previously mentioned, ambient light is also a key factor affecting the accuracy of smart phone imaging. Generally, with increasing illumination, more photons are reflected from the subject and reach the camera sensor, potentially leading to overexposure. Settings in the camera (including ISO, shutter speed and aperture value) are thus adjusted automatically to control the exposure. To evaluate the practicability of our SCQP, the performance of our assay was analyzed under different illumination conditions (camera settings of ISO and shutter speed with/without automatic adjustment). A linear relationship was found between the illumination change and the quantitative color characteristics, such as S and G/V (Fig. S16), indicating that the correction of the color value under different illumination conditions can be achieved through a simple linear formula. In general, the results obtained by our app (with/without automatic adjustment) were comparable with those from the UV–Vis spectrometer under varying ambient illumination (Fig. S13b, c, Table S8 for camera settings).

Robustness and compatibility. The robustness and compatibility of our mathematical and algorithmic methods, which were integrated within a tailor-made app, were evaluated. Generally, the differences in hardware, system and internal calibration methods of different smartphones and/or operating systems/software may result in two problems for SBC analysis. First, the color values of the same sample obtained by different smartphones may be different. Moreover, the same image could lead to different color values using different smartphones, as a discrepancy in color interpretation [1].

To test this, three commercial brands of smartphones were used to image the same emodin sample, and the program ImageJ [2] was applied to assess our app, with results shown in Fig. S17. The fitted lines of the three smartphone brands had only small differences (mean |error| = 4.46 (Brand A), 4.76 (Brand B), 5.09 (Brand C) μg mL^{-1} respectively), with the dense distribution of the data points however indicating that our app had satisfactory compatibility across the brands of smartphones tested (Fig. S17a-c).

To investigate the cross-platform versatility, the sample image was taken by two types of smartphones with different operating systems (Brand A and B). The image taken by Brand A was transmitted to a computer with the Windows 10 operation system (for reference) and then ImageJ and our app (installed on Brand A smartphone) were used to extract image color values (see Fig. S18). The results revealed no difference in color interpretation and quantitative results (Fig. S18c, mean |error| = 0.49 μg mL^{-1}). Upon the basis of the above validation, the image taken by Brand B was transmitted to another smartphone (Brand A) and the computer again. Subsequently, ImageJ

[2] and our app (installed on Brand A smartphone) were applied to extract the color values of the image. The results indicated that the quantitative results of our app remained stable despite discrepancies occurring in the color interpretation of the same image by the two types of smartphones (Fig. S18a, mean |error| = 1.56 μg mL^{-1}; Fig. S18b, discrepancies of color interpretation, Fig. S18d, e validation flow).

To further explore the applicability of SCQP, we used a colorimetric enzymatic assay to detect glucose in solution. The reaction yields either a blue color (TMB reaction – see methods) or a yellow color (when the reaction is stopped by an acid solution, Fig. S19) [3]. When compared with the traditional colorimetric quantitative parameter (QP), more stable and precise performance was provided by SCQP (Fig. S19a-d). For glucose detection (blue state), mean |error| (SCQP) and mean |error| (QP) were 0.08 and 0.16 μg mL^{-1}, respectively. Similar results were observed in glucose detection in the yellow state, in which the mean |error| (SCQP) and mean |error| (QP) were 0.076 and 0.083 μg mL^{-1}, respectively. These results showed that the proposed SCQP can be used universally for colorimetric analysis under ambient light with good performance and potential for clinical applications.

Supplementary Methods

Detection processes. Samples were dispensed in the grooves of the chip. An illuminometer was placed on the side of the sample to record the illuminance value during photographing. We cropped the sample image from the real-time imaging or smartphone album in the app, and the image color or sample concentration values were automatically extracted, calculated and displayed. These data were then used to fit linearity or accomplish quantification (by clicking the "CALIBRATION", "QUANTITATIVE" buttons, respectively). The UV–vis spectrophotometer [Shimadzu, UV-2600] results were used for the standard results. Smartphone brands were iPhone 8, Huawei P20 Pro and Vivo IQOO in all the experiments, although we chose to display the results as brand A, B, C to unlink the results from the brands.

SCQP. Equations S1 and S2 define S and V. Taking emodin as an example, two color spaces (RGB and HSV) are connected by Equation S3. L_{max} and L_{min} are the upper and lower limits of concentrations can be detected by the instrument, respectively. However, the nonlinear results Equation S5 of S were due to the changes in Equation S4.

$$S=\begin{cases}\dfrac{Max\ (R,G,B)\text{-}Min(R,G,B)}{Max\ (R,G,B)} & V\neq 0\\ 0 & V=0\end{cases} \tag{S1}$$

$$V=Max\ (R,G,B) \tag{S2}$$

$$S=\begin{cases}1\text{-}\dfrac{Min(R,G,B)}{V} & V\neq 0\\ 0 & V=0\end{cases} \tag{S3}$$

Example: Emodin

$$\begin{cases}Min(R,G,B)=G,\ \ Max(R,G,B)=R & c\leq L_{Max}\ (V\neq 0)\\ Min(R,G,B)=B,\ \ Max(R,G,B)=R & c\rightarrow L_{Min}(V\neq 0)\end{cases} \tag{S4}$$

Finally

$$S=\begin{cases}1\text{-}\ \dfrac{G}{V} & c\leq L_{Max}\ (V\neq 0)\\ 1\text{-}\ \dfrac{B}{V} & c\rightarrow L_{Min}\ (V\neq 0)\\ 0 & V=0\end{cases} \tag{S5}$$

Where c is the concentration in the sample.

Optimization of the edge segmentation algorithm and signal extraction. To optimize the smartphone performance and implement the algorithm with minimal computational resource cost, a grayscale copy of the raw image was created with the same size. The final result of the algorithm was presented as a raw image.

We developed two methods that can extract image color values. The first involved edge segmentation using a feature extraction method customized for our chip (using the global boundary scan algorithm). Specifically, by scanning the overall color difference between the sample and the chip, the boundary position was quickly obtained, and the area out of the sample was filtered by calculating the ratio of global area pixels. Subsequently, the average color values of the sample area were calculated.

Alternatively, to measure the color values without using a chip, edge detection technology based upon the Sobel algorithm[4] was used. Specifically, Sobel convolution of the raw image provided the

edge discrepancies of each pixel in the X and Y directions and the image segmentation was further completed by setting the manual threshold obtained by a priori knowledge (Fig. S20). The center point of the sample area was then calculated, and an extended ROI for average color value measurement was drawn. Taking emodin detection as a model, six different ROI sizes were explored (10×10 pixels, 30×30 pixels, 50×50 pixels, 80×80 pixels, 100×100 pixels and 150×150 pixels, Fig. S21), and a size of 30×30 was ultimately chosen as the best ROI size for our program (customized ROI is also supported in our app).

In addition, our program also supports touch color picking. If the user clicks on the area of interest in the image on the screen, the program will automatically extend this position as the center to form an ROI and automatically read color values.

Sample preparation. Detailed information about reagents is presented in Table S9. The green ink was from shin-ryoku. In a 25 mL volumetric flask, 2.5 mg of emodin was accurately weighed and dissolved into (2.7 M NaOH:2 M NH_4OH=1:1 (v/v)). 0.1 mg of glucose oxidase was added to 100 µL of glucose standard solutions with different concentrations, and the mixture was incubated for 10 min at 37 °C to obtain solution A. 0.05 mg of horseradish peroxidase and 50 µL of trimethylbenzene (TMB) solution (3.2 mM) were added to solution A, diluted to 1 mL by adding acetate buffer (pH = 5), and stored in the dark for 30 min at 37 °C leading to a blue color, solution B. 500 µL of 0.1 M sulfuric acid was added to an equal volume of solution B to end the reaction, turning it yellow.

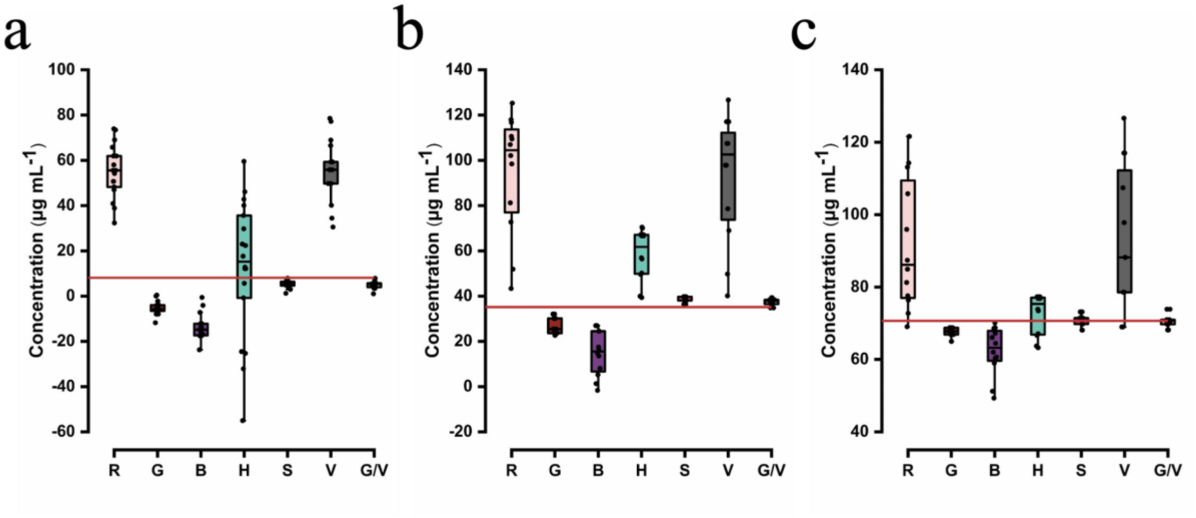

Fig. S1. Visualization of the analyzed output for each quantitative parameter. a-c, a, b and c represent the results of samples S1, S2 and S3 corresponding to low, medium and high concentration of emodin, respectively (n=18 independent measurements for each). The horizontal line represents the results obtained using a UV–Vis spectrophotometer (mean |error| (G) = 8.40 µg mL^{-1}, mean |error| (G/V) = 2.27 µg mL^{-1} and mean |error| (S) = 2.46 µg mL^{-1}).

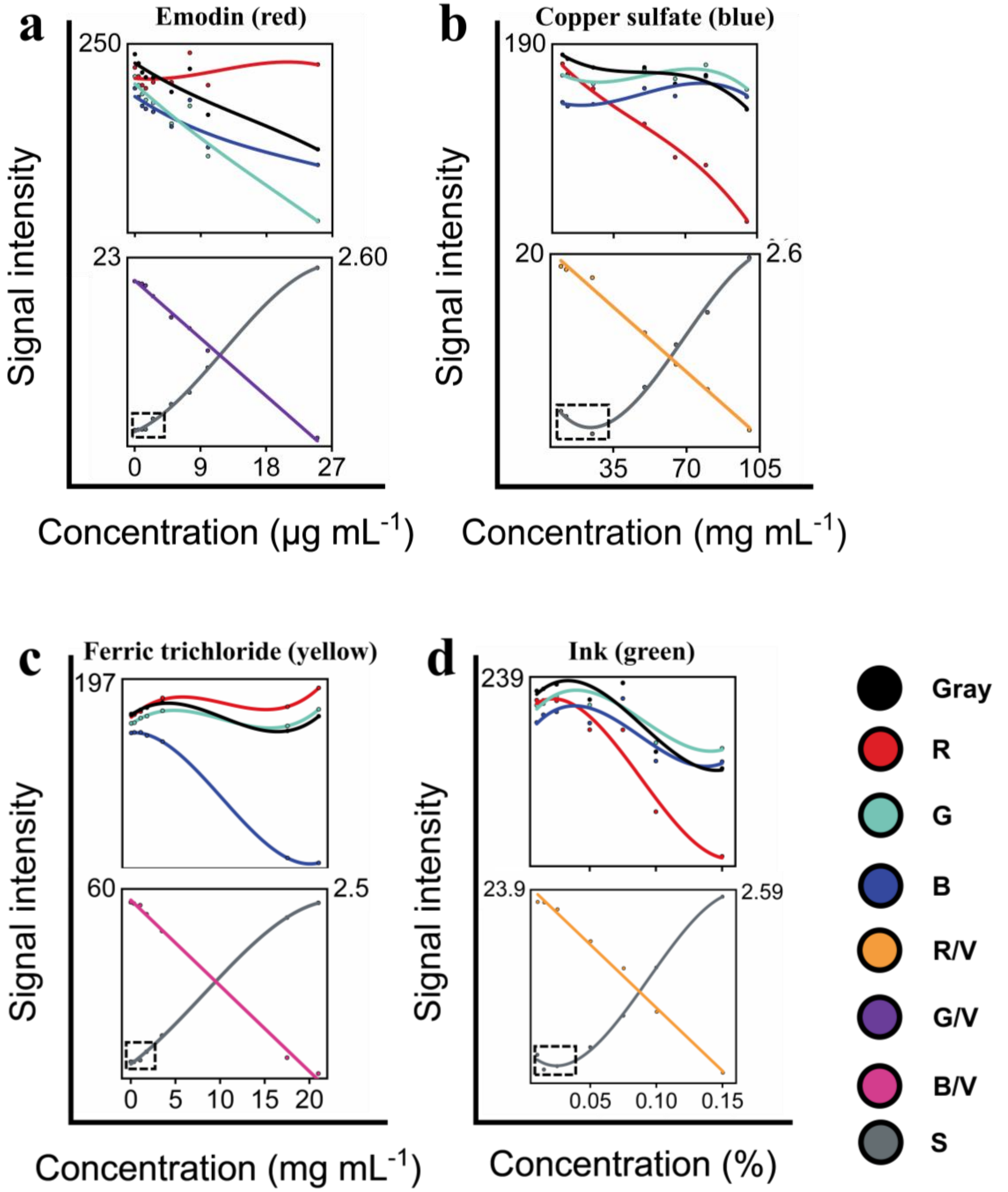

Fig. S2. Validation of S boundedness in different color samples (dashed square indicate the non-linear region of S parameter): a, Emodin (red). b, Copper sulfate (blue). c, Ferric trichloride (yellow). d, Ink (green). X-axes have different scales due to the different analytes.

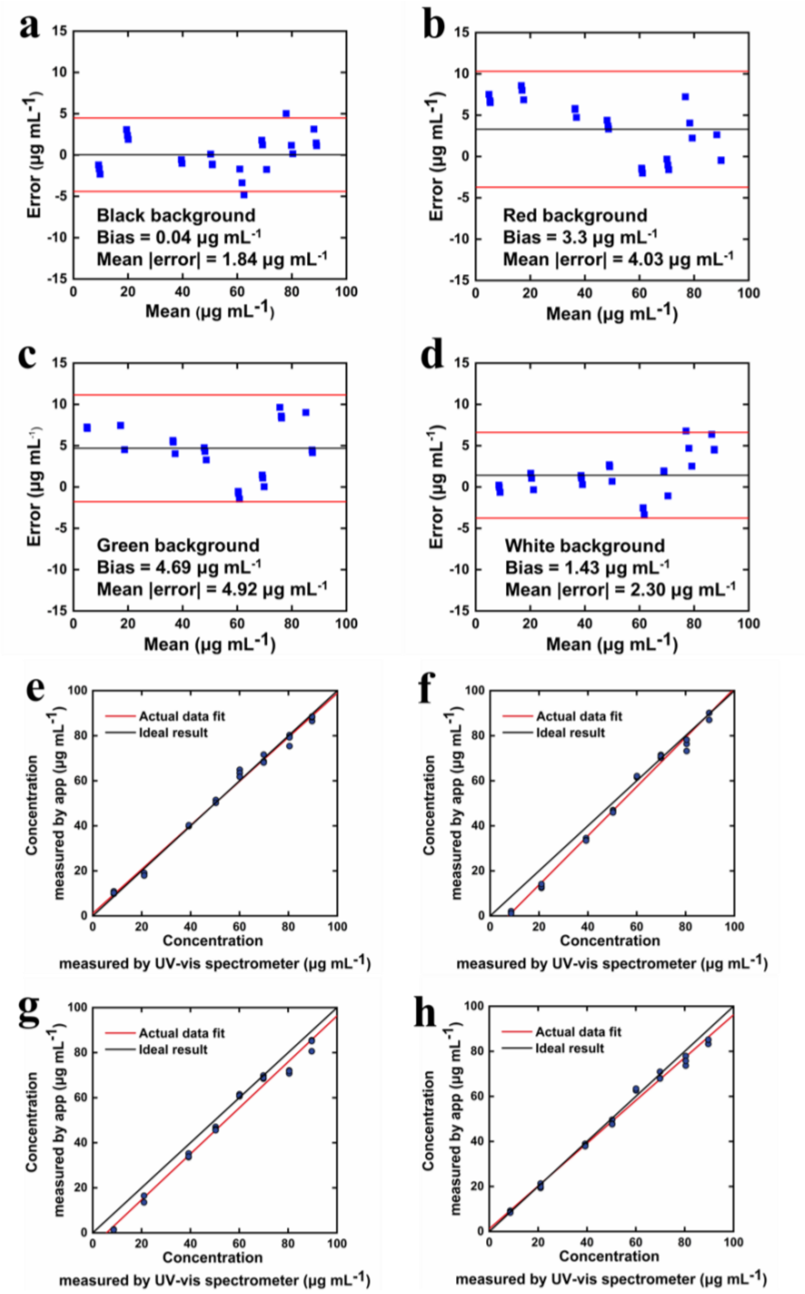

Fig. S3. Comparison of quantitative results in different backgrounds. a-d, Bland–Altman analysis. The red lines are 95% LoA (black: +4.48, -4.40 μg mL^{-1}, white: +6.61, -3.76 μg mL^{-1}, red: +10.31, -3.72 μg mL^{-1}, green: +11.15, -1.78 μg mL^{-1}), which embodies the overall discrepancy boundary between the results from our app and UV–Vis. The Y-coordinate and X-coordinate represent the error (result(UV) – result(app)) and average value of the quantitative result measured by the app and UV–Vis, respectively. The bias is represented by black lines, which reflect the average error level of the two methods (black: 0.04 μg mL^{-1}, white: 1.43 μg mL^{-1}, red: 3.3 μg mL^{-1}, green: 4.69 μg mL^{-1}). e-h, Regression analysis was used to evaluate the quantification of the results, with the red solid lines representing the actual data fit, and the closeness to the ideal result (the black line) reflecting the performance of our system under different backgrounds, in which we note that the non-linearity of G/V arising at low concentrations (see Figure S2) leads to decreased performance in f & g. The samples used are presented in Supplementary Figure S4.

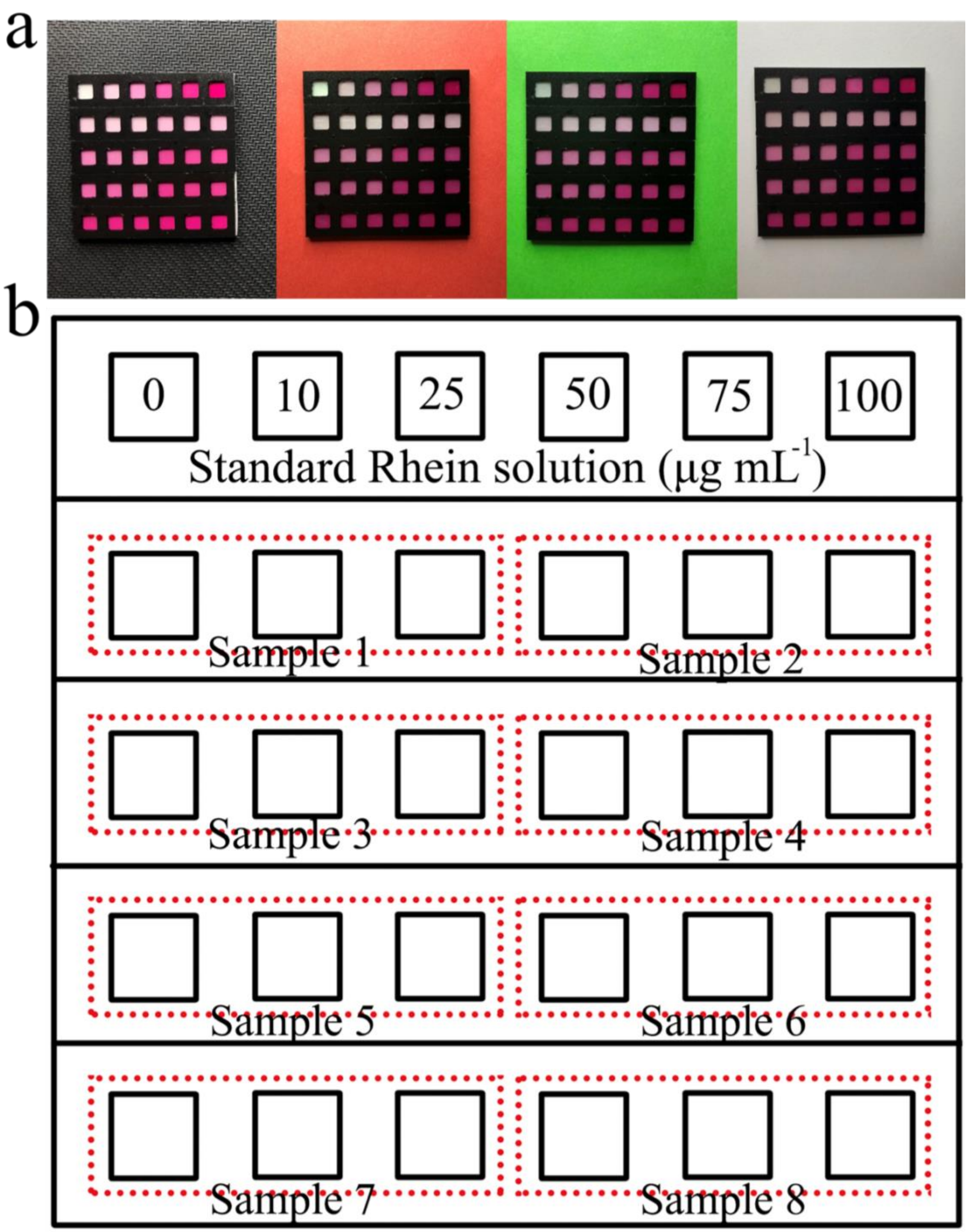

Fig. S4. a, The sample images with different backgrounds. Each sample were measured in triplicate (Illuminance: 662 Lux, shooting distance: 11 cm, camera settings are automatically adjusted). b, Sample identification.

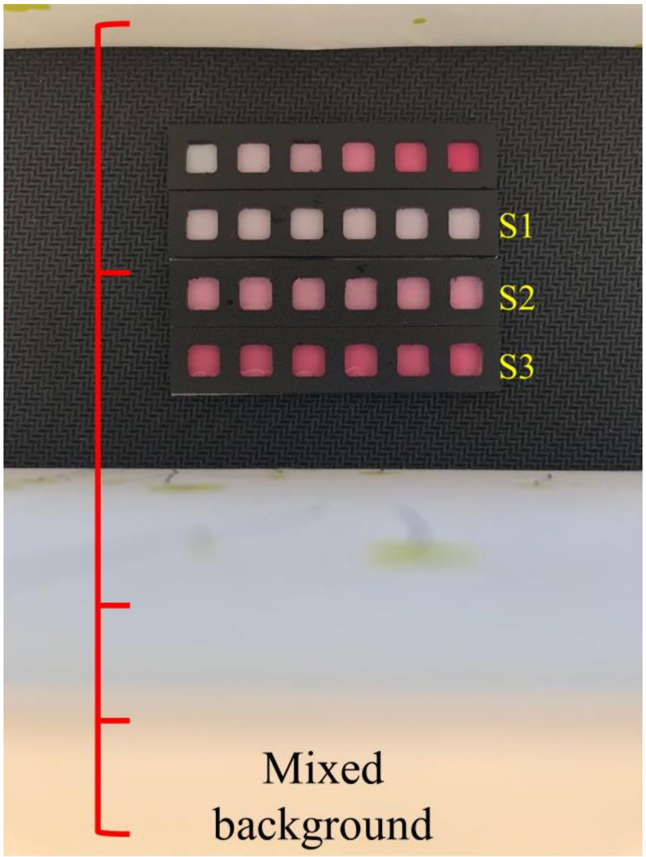

Mean |error| (G, μg mL^{-1})	Mean |error| (G/V, μg mL^{-1})	Sample
12.29 (±2.52, n=6)	2.63 (±0.9, n=6)	S1
12.42 (±4.68, n=6)	1.68 (±1.72, n=6)	S2
5.82 (±2.26, n=6)	1.62 (±2.52, n=6)	S3

Fig. S5. Verification of the performance of our system on a mixed and uneven background in which brown, black, white with random green and gray dots, and dull and light yellow colors were embedded. (a) The analyte in first line shows the standard Rhein solution (0, 10, 25, 50, 75, 100), and S1, S2 and S3 are samples with low, mid and high concentrations respectively. (b) Table comparing our corrective strategy (G/V) and no corrections (G), with reference to UV–Vis measurement (ground truth).

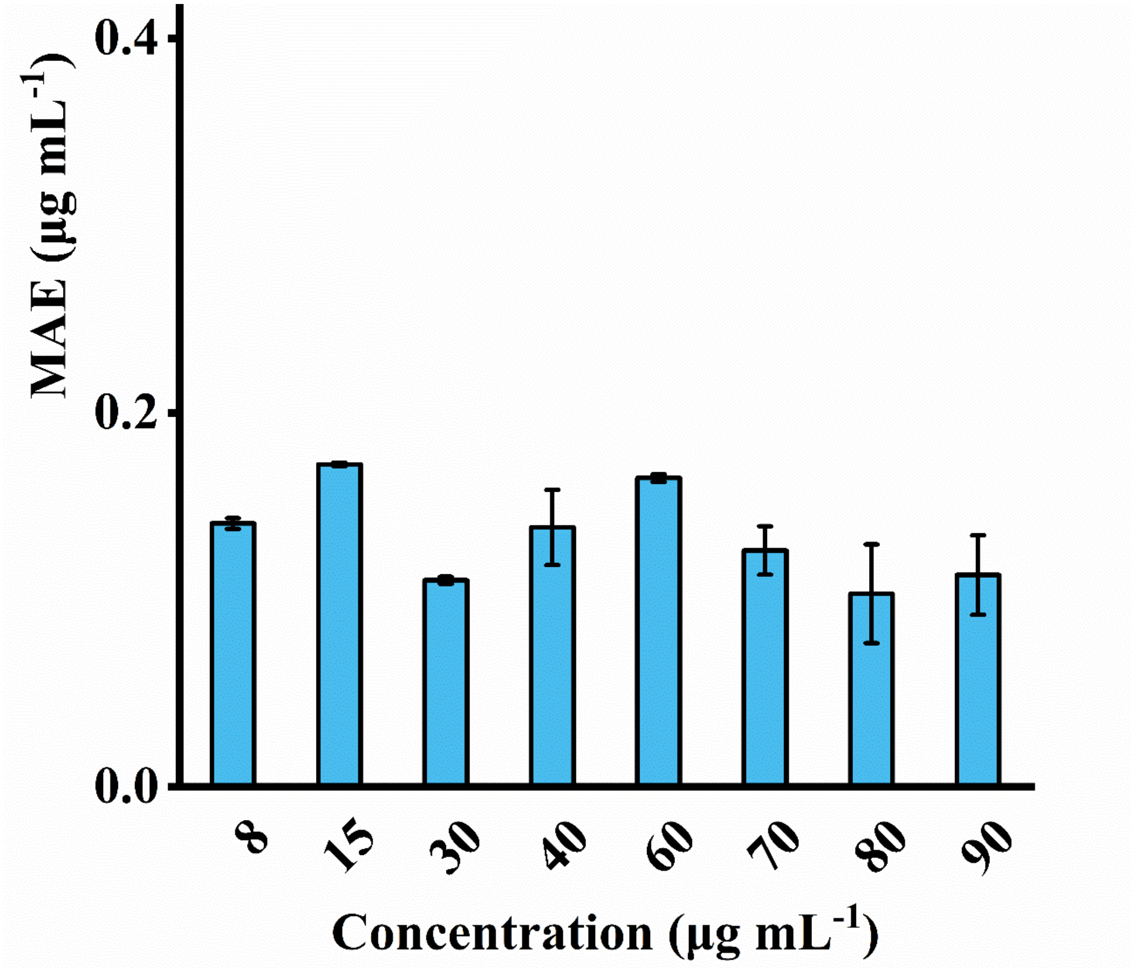

Fig. S6. Mean absolute error (MAE) of emodin samples measured over different surfaces of backgrounds (with a black background as reference).

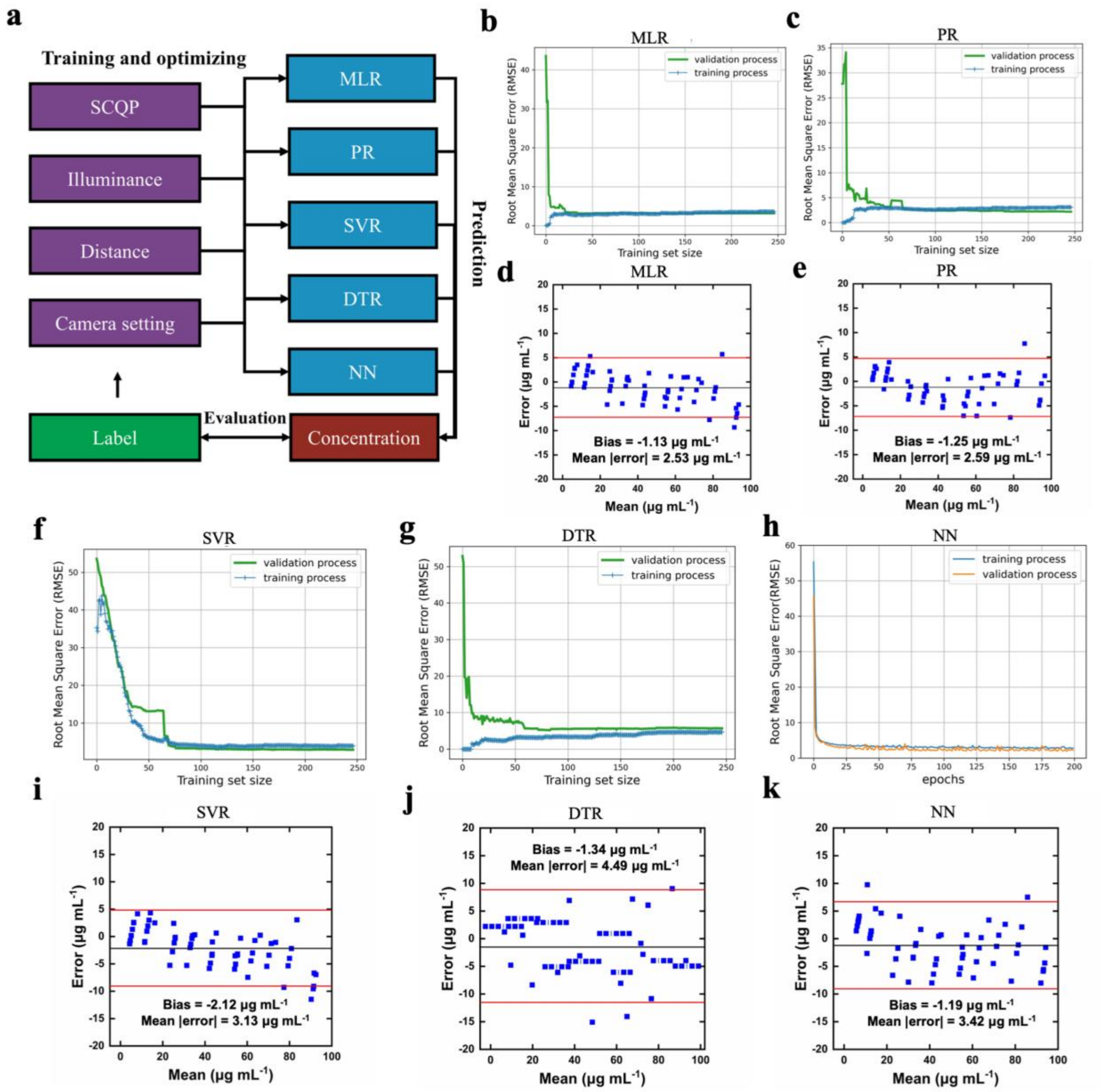

Fig. S7. Visualization of the training process using learning curves for five employed strategies (**a**, workflow. **b, c**, **f-h** overall 310 sets of training data, 20% was split as validation set) and prediction performance of the trained models in 60 real-time imaging samples (**d, e, i-k**). The sample concentrations are over range 0-100 μg mL^{-1}, and were split in 10 decade-intervals with 6 concentrations in each range including ranges outside of the labelled classes, as well as random environment conditions.

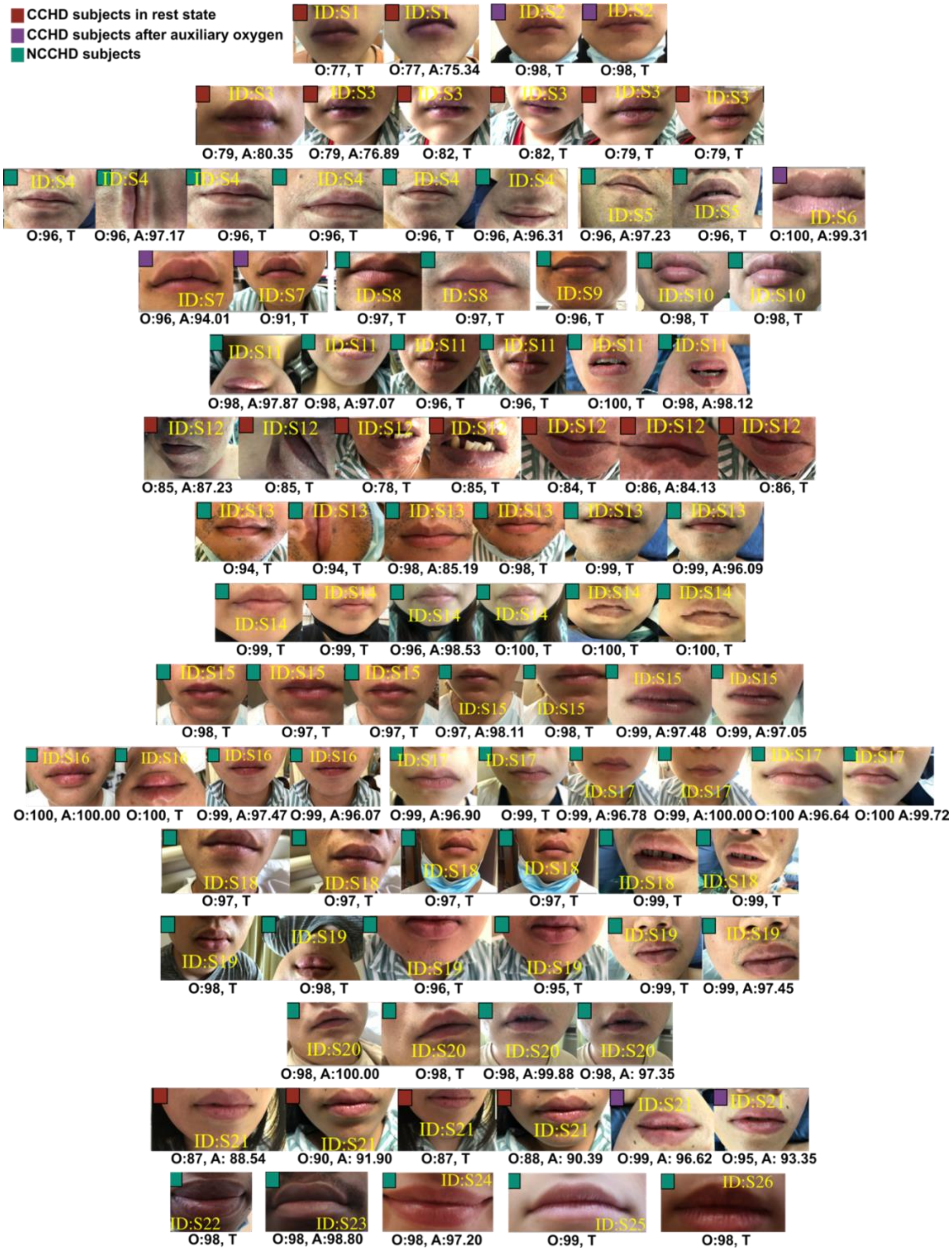

Fig. S8. Image set of clinical samples, for congenital heart disease patients (CHD) suffering from cyanosis (CCHD), with (purple squares) and without oxygen supply (red squares), as well as patients with no cyanosis symptom (NCCHD, green squares). The labeling underneath the image indicates the oximetry measurement (O) in %, "T" identifies training images, whilst the measurement for the app ("A") is also provided in %. Linked data is presented in Supplementary Tables S3-S5.

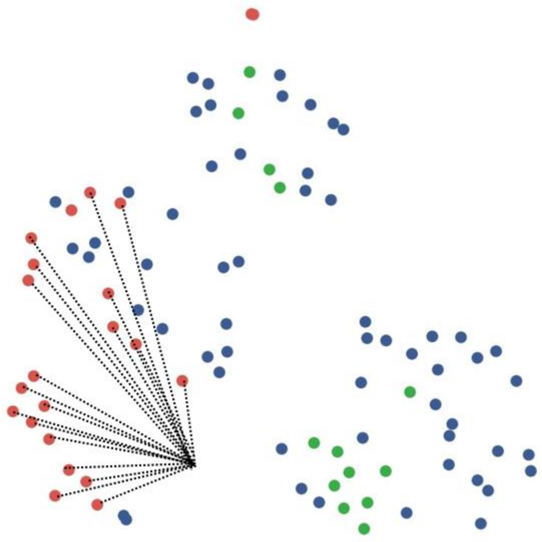

Fig. S9. T-SNE clustering of patient samples (red, SpO_2 < 95%, green, 95% ≤ SpO_2 < 97%, blue, 97% ≤ SpO_2 ≤ 100%), indicating the monitoring and warning ability of our app to SpO_2 level.

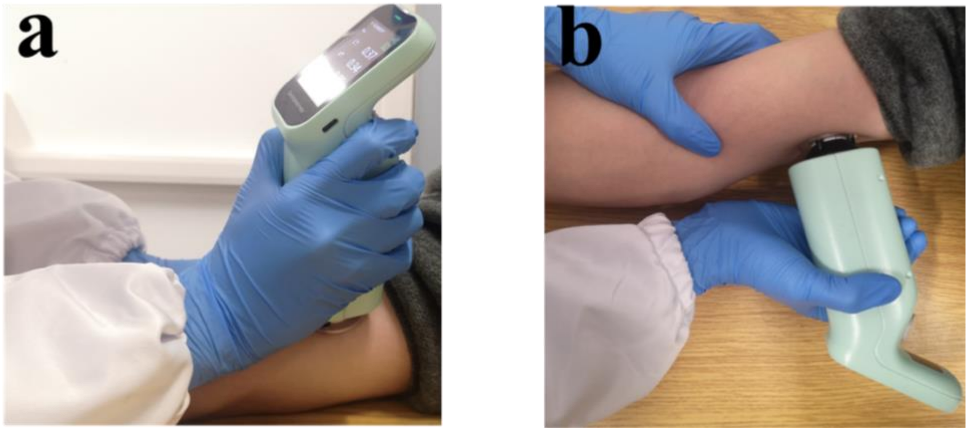

Fig. S10. Skin measurement using a spectrophotometer on four parts (left and right upper outer – a- and inner – b- arms).

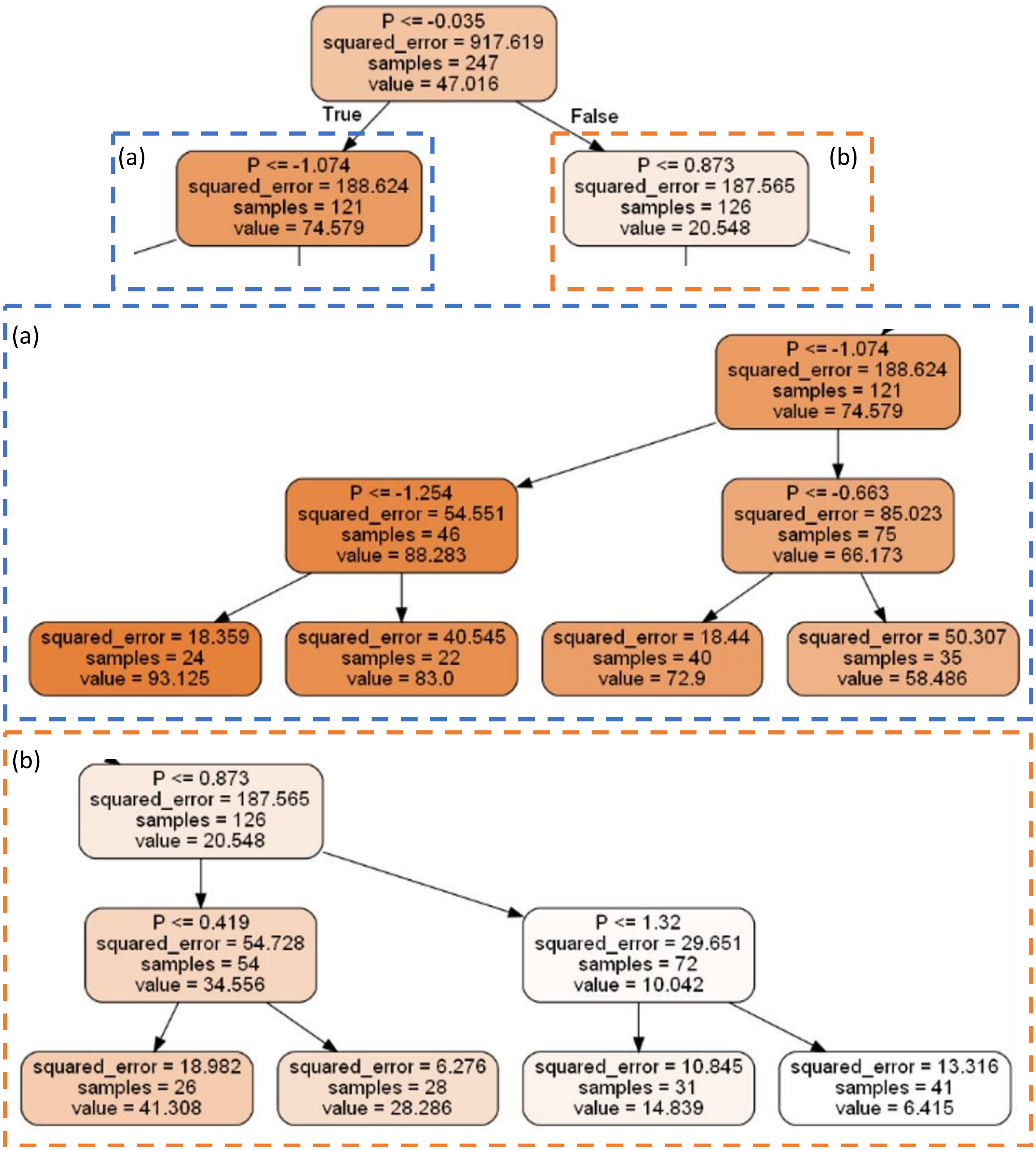

Fig. S11. DTR model architecture. P and samples present the classification threshold and the number of analytes under decision region of trained DTR model. Squared_error and value are predicted MSE and result of trained DTR model respectively. The figure has been broken up in sub-panels a-b to increase readability.

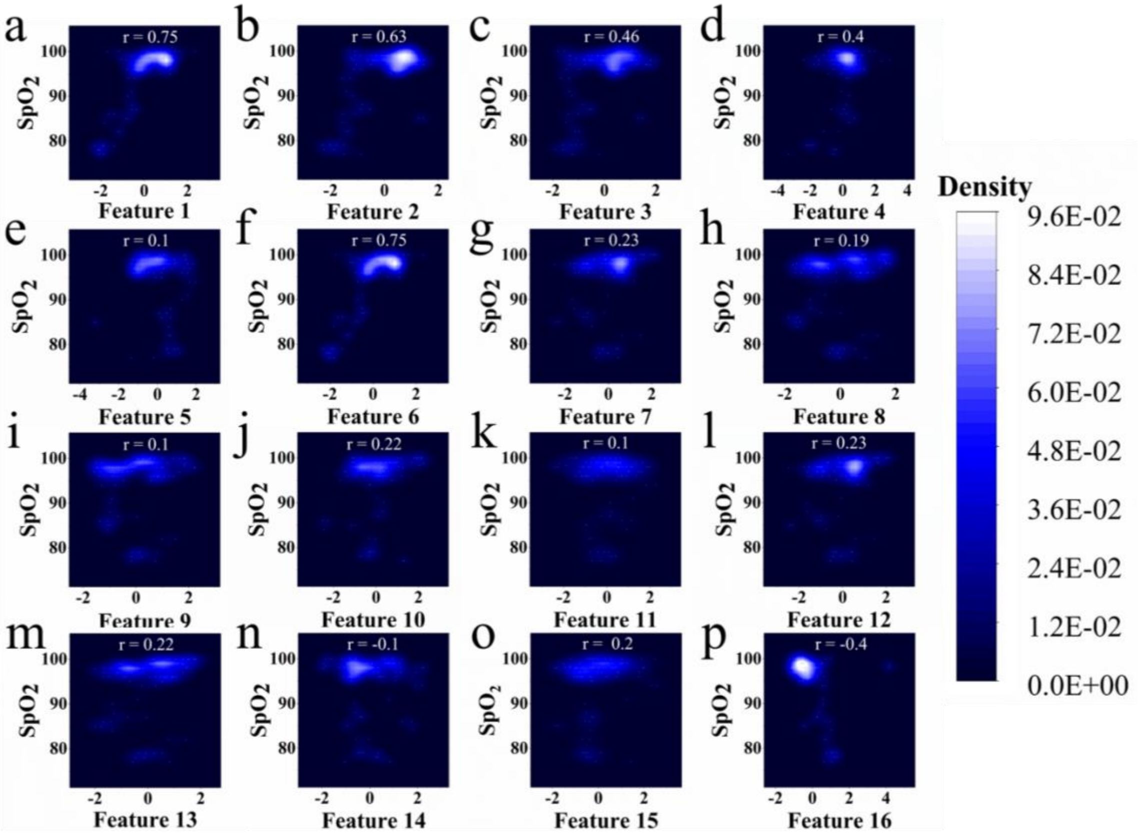

Fig. S12. Pearson correlation (r) coefficient of each key feature against SpO_2 level in our 16-dimenssional features. Y-axis represents SpO_2 levels measured by gold-standard blood gas test, and feature 1-16 indexes the mined features from lip image metadata. Features have been normalised by z-score. Color bar represents the distribution of data in density range of 0 to 0.1.

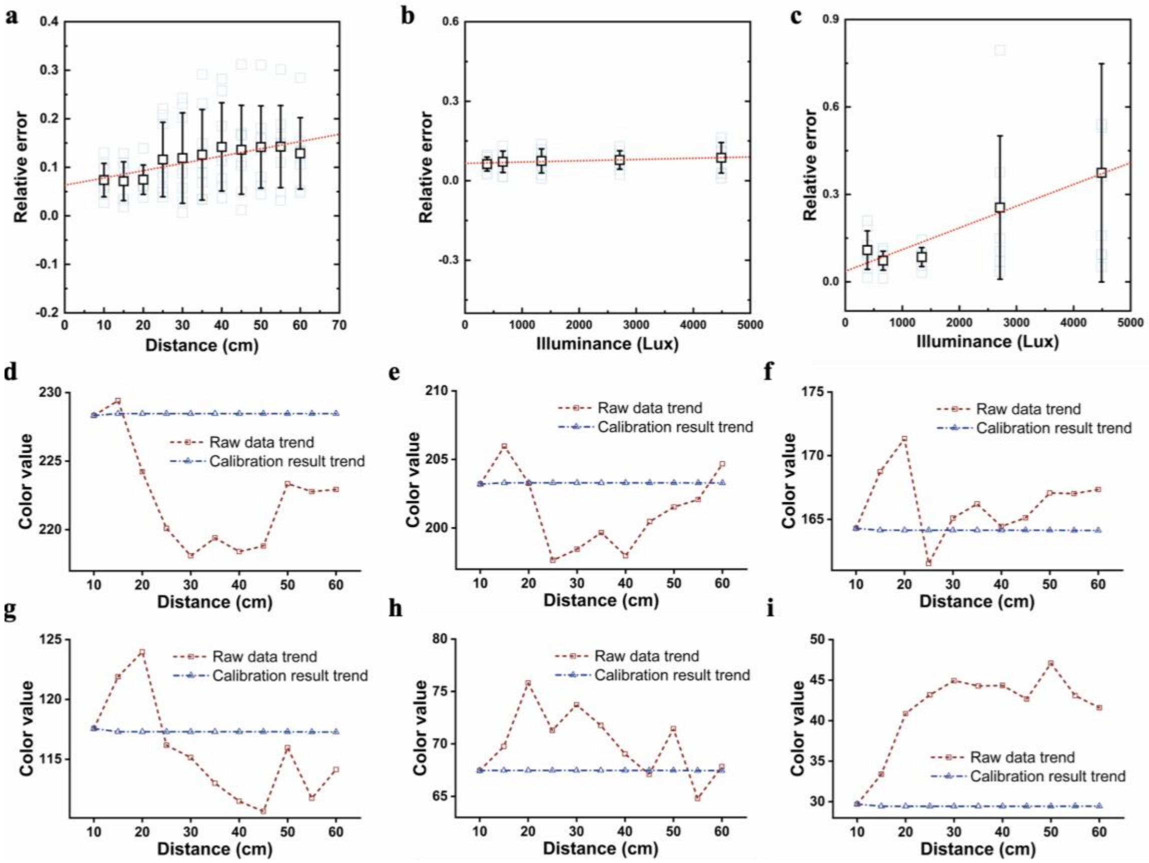

Fig. S13 Influence of different shooting distances and illuminances on system quantification. Eight groups of emodin samples were measured in triplicate. **a,** Dashed red lines represent the regression line fitting between the average error point (white square) and shooting distance, indicating the stable performance of the app under different distances (the blue square is the data point, illuminance: 662 Lux, black background, shooting distance selected in a range from 10 cm to 60 cm, with a 5 cm increase every step. ISO: ISO-320, shutter time: 1/60 s, aperture-value: f/1.8 and other camera settings kept default). b-c, Working performance of the app under different illumination conditions. **b,** Default (automatic) camera setting mode. In (a) and (b), the dashed red line represents the regression line fitting between the average error point (white square) and illuminance, and the blue square is the relative error distribution under each illuminance. **c,** Camera manual setting mode (without automatic adjustment). Partial error was generated owing to overexposure in high illuminance (shooting distance: 11 cm, illuminance: 386, 662, 1340, 2710, 4490 Lux, aperture-value: f/1.8, shutter time: 1/60 s, ISO: ISO-320). **d-i**, Performance of the iterative correction algorithm. Six representative samples were tested by the algorithm (0, 10, 25, 50, 75, 100 μg mL^{-1}). Each sample was shot at N cm (N = 10, 15, 20, 25, 30, 35, 40, 405, 50, 55, 60 cm), and then the iterative correction was performed in each sample from M cm to a distance (here, we selected 10 cm as an example) at 5 cm intervals (for example, from 20 cm to 15 to 10 cm, M = 15, 20, 25, 30, 35, 40, 45, 50, 55, 60 cm).

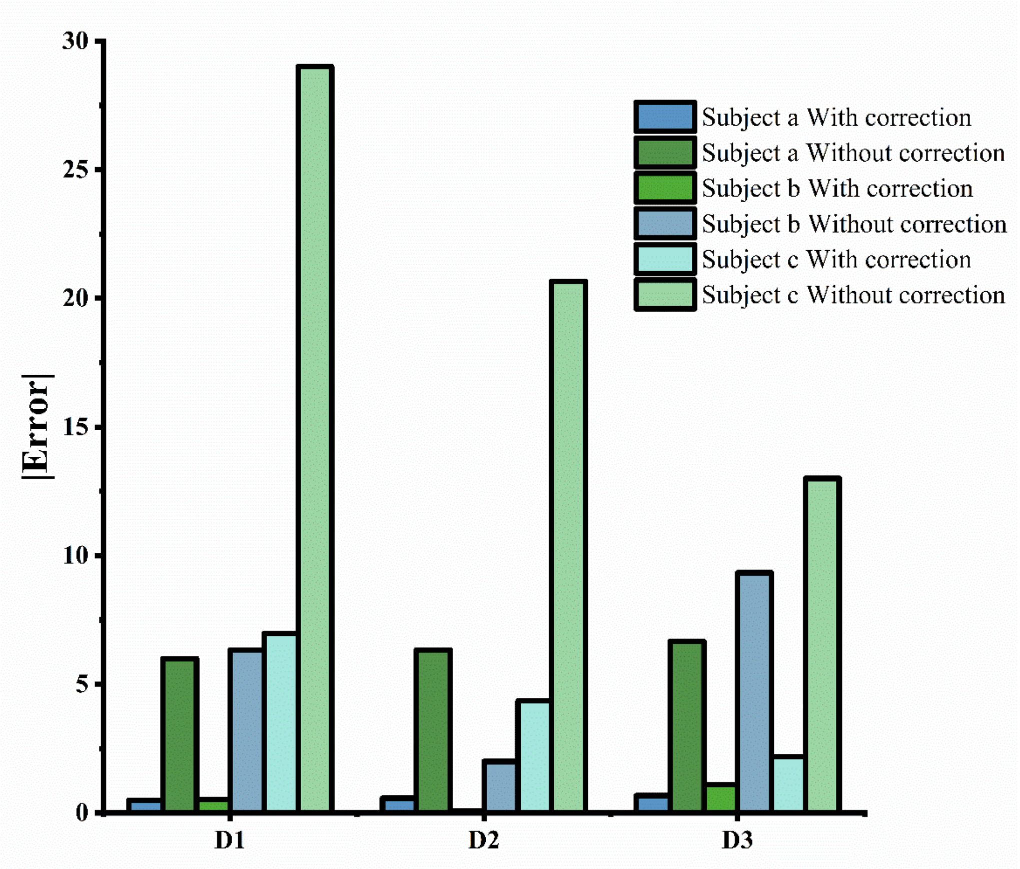

Fig S14. Performance of the iterative correction algorithm on three subjects. D1, D2 and D3 represent error correction between distances of (10, 15), (15, 20) and (20, 25). |Error| represent error of color values between two distances (take R value as an example).

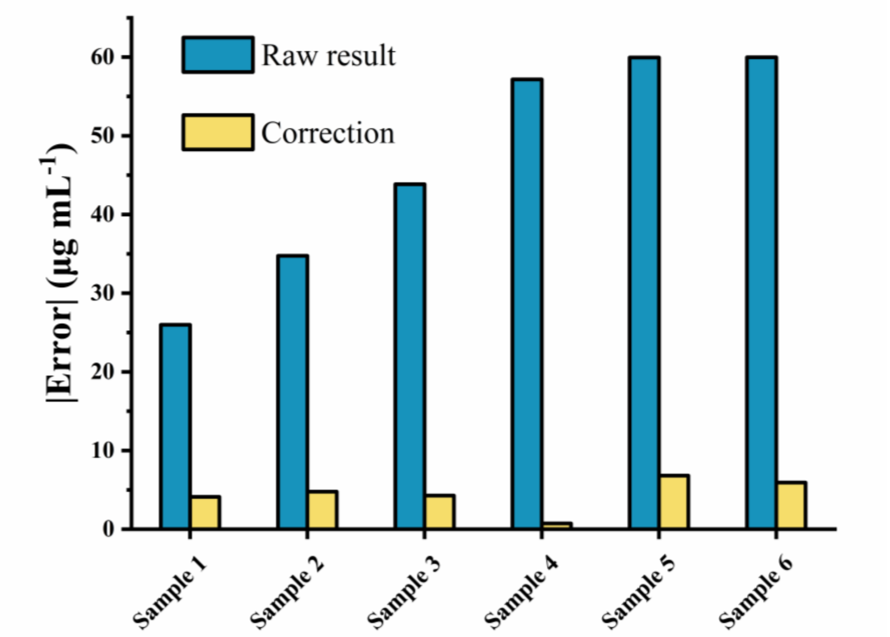

Fig. S15. Performance of the iterative correction algorithm on image taken with automatic camera settings.

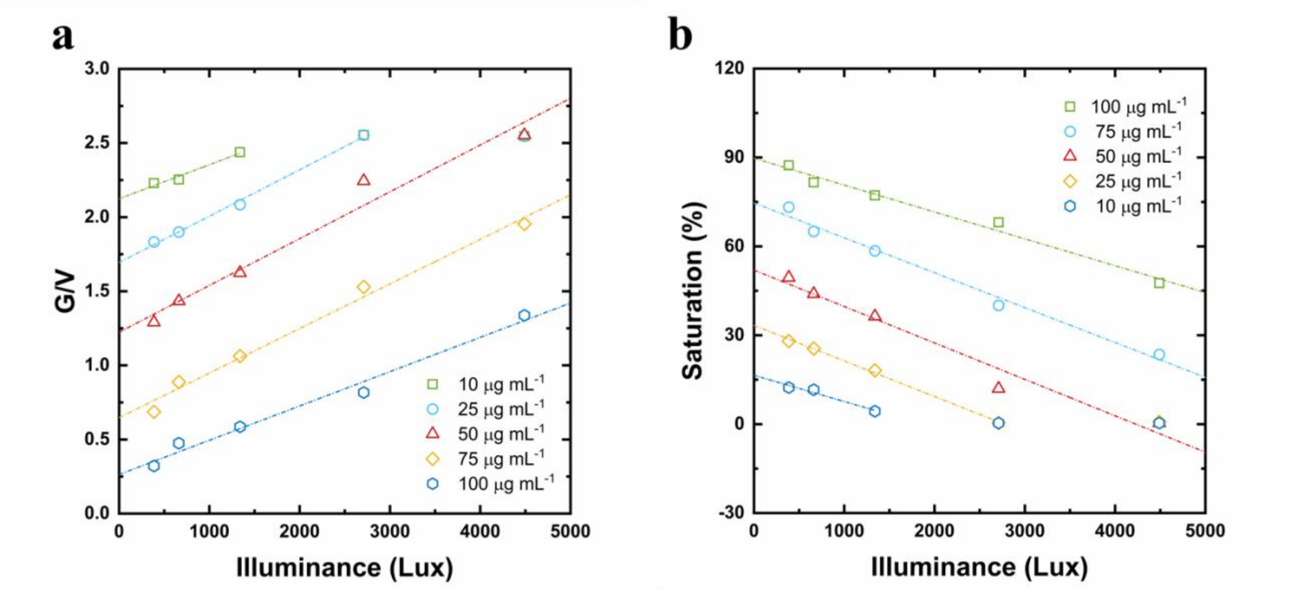

Fig. S16. Relationship between the color value and illuminance for different concentrations of emodin from 10-100 μg mL^{-1}). a – G/V; b – saturation). The unconnected part (samples of 10 μg mL^{-1} and 25 μg mL^{-1} under high illuminance) exhibited a trend shift owing to overexposure.

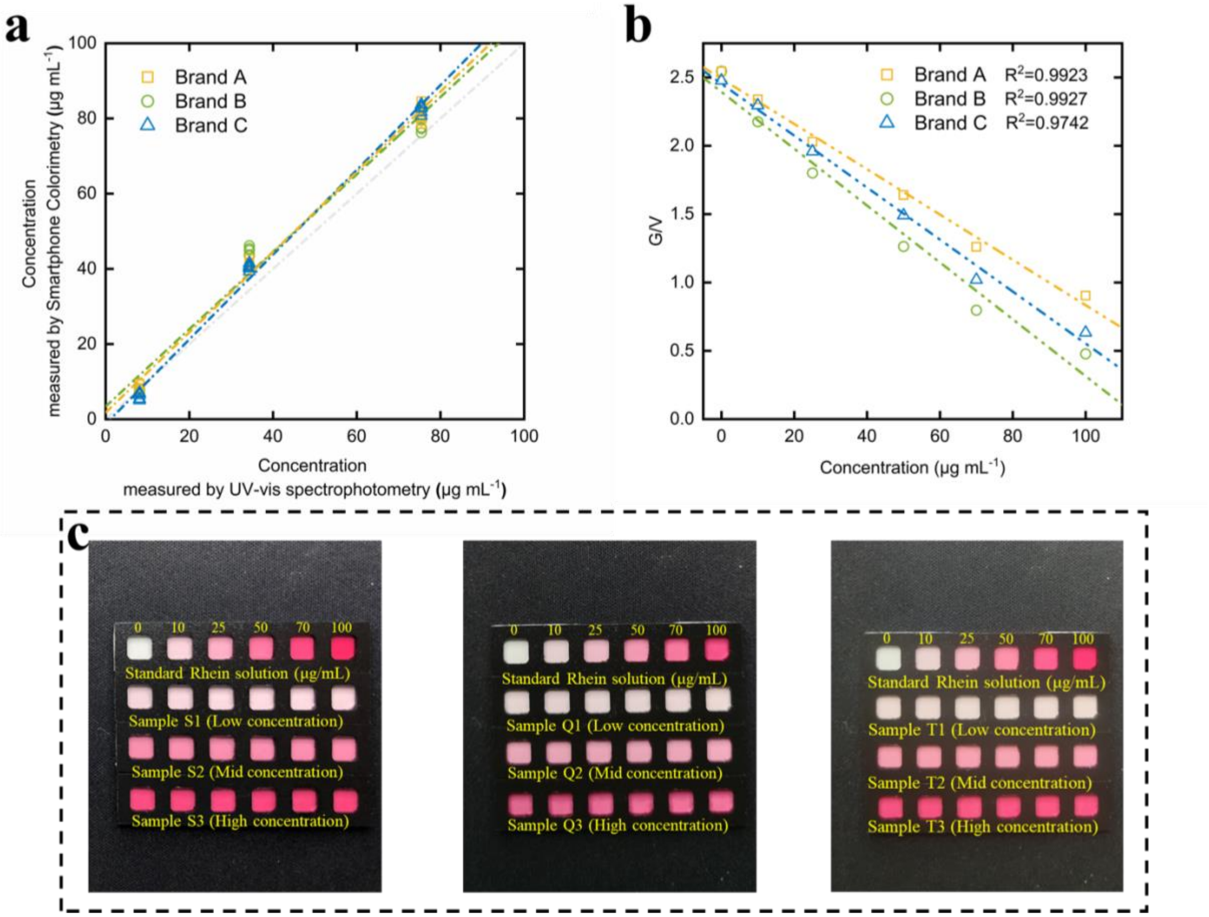

Fig. S17. Verification of the compatibility of the developed app using different smartphones. (a) The app result generated using three brands of smartphones. (b) Quantitative curve of emodin in three brands of smartphone. (c) Sample images taken by three different smartphones (smartphone brand: A, B and C from left to right, Illuminance: 662 Lux, Distance: 11 cm, camera setting are automatically adjusted).

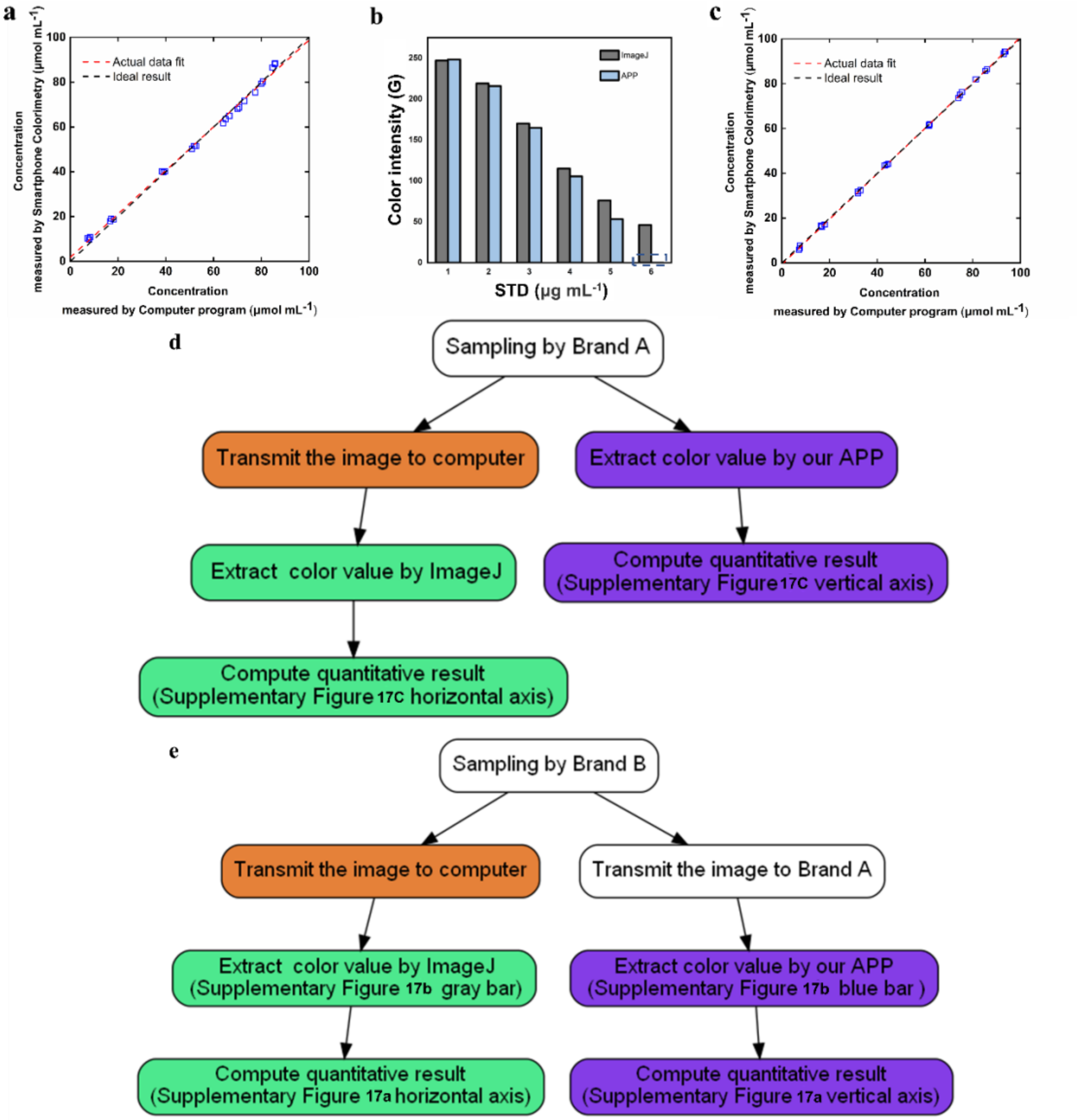

Fig. S18. Verification of the robustness of the developed app for different devices. (a) The quantitative result in two devices with different color interpretation. Dashed black line represents ideal result (the result obtained in smartphone equals that obtained in computer program). Dashed blue line represents an actual measurement (with the workflow showed in d). (b) Discrepancy of color interpretation. The color value was close to 0 at dashed blue square. STD is standard sample solution. (c) The App result compared with standard reference (the workflow showed in e). All samples were measured 3 times, independently. (d), (e) The workflow of the two validation strategies.

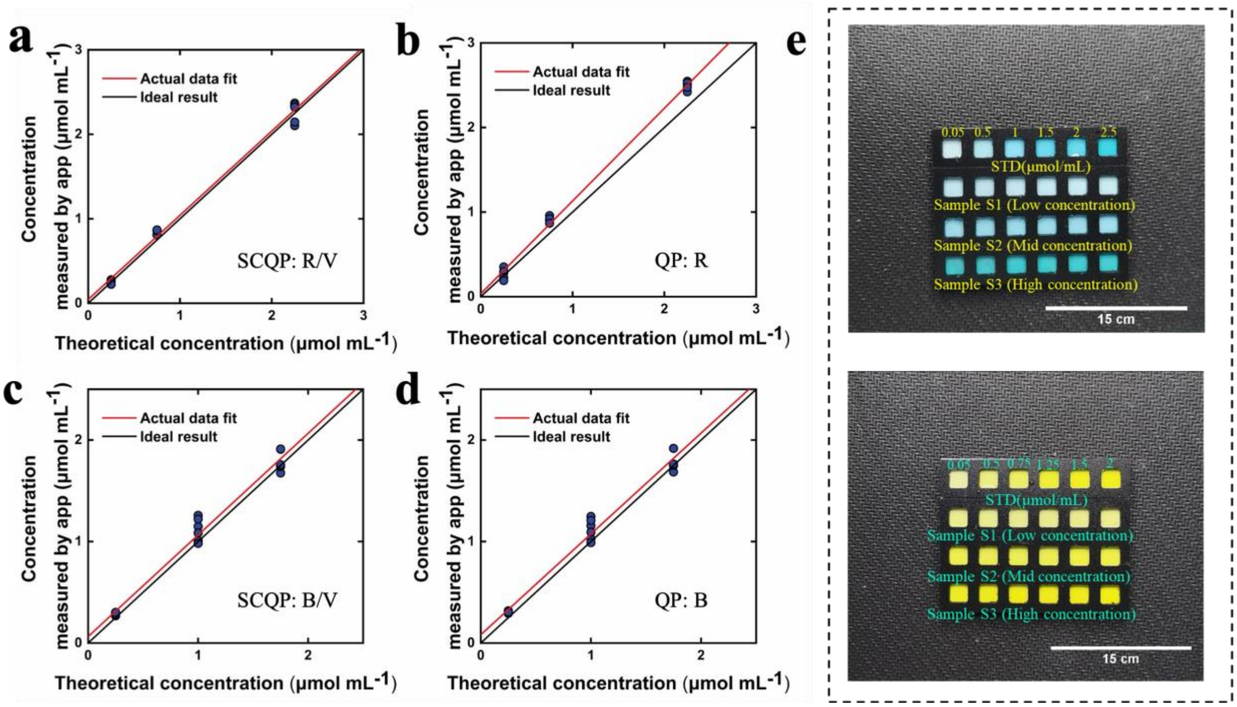

Fig. S19. Verification of the system applicability to a biochemical assay. Compared with the traditional quantitative parameter (QP), the improved working performance of our SCQP is represented in two other samples of different colors (a-d). The black lines represent ideal result (45-degree line), whilst the red lines represent actual detection result fit. Points represent detection result error between app and standard. e, Sample image, for glucose detection with different color states (blue - top and yellow - bottom).

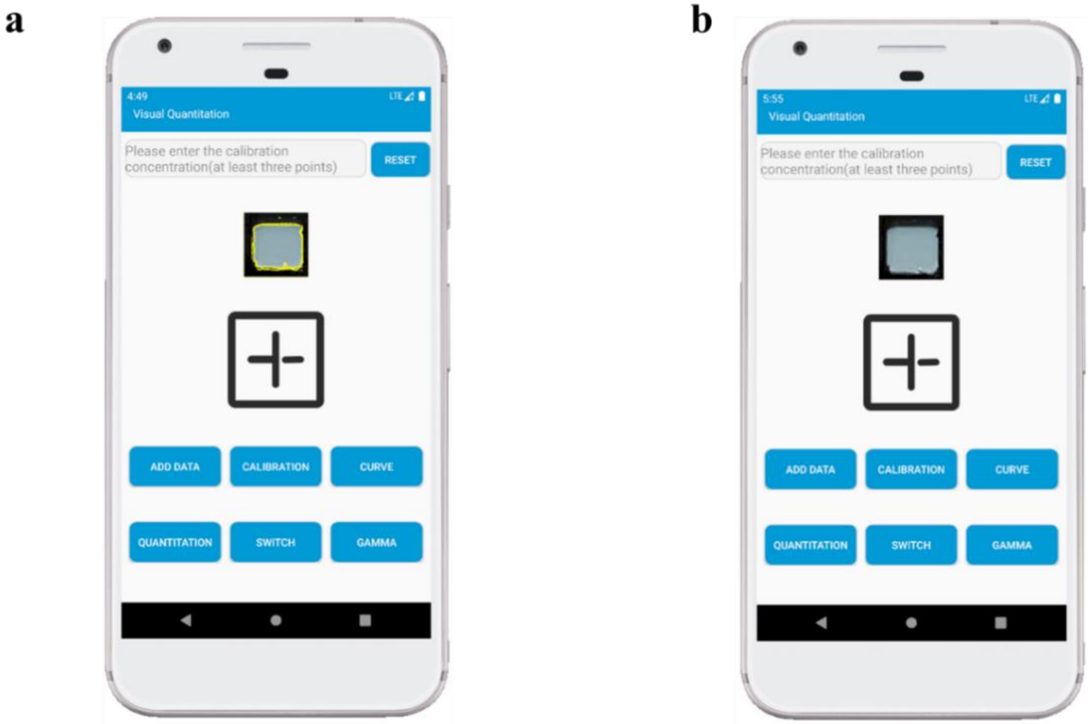

Fig. S20. Edge Segmentation. Visualization of (a) edge segmentation algorithm and (b) raw program. The image edge was automatically segmented by applying our algorithm. Using Pixel XL in Android Studio (Google, Mountain View, CA) as demonstrated device model.

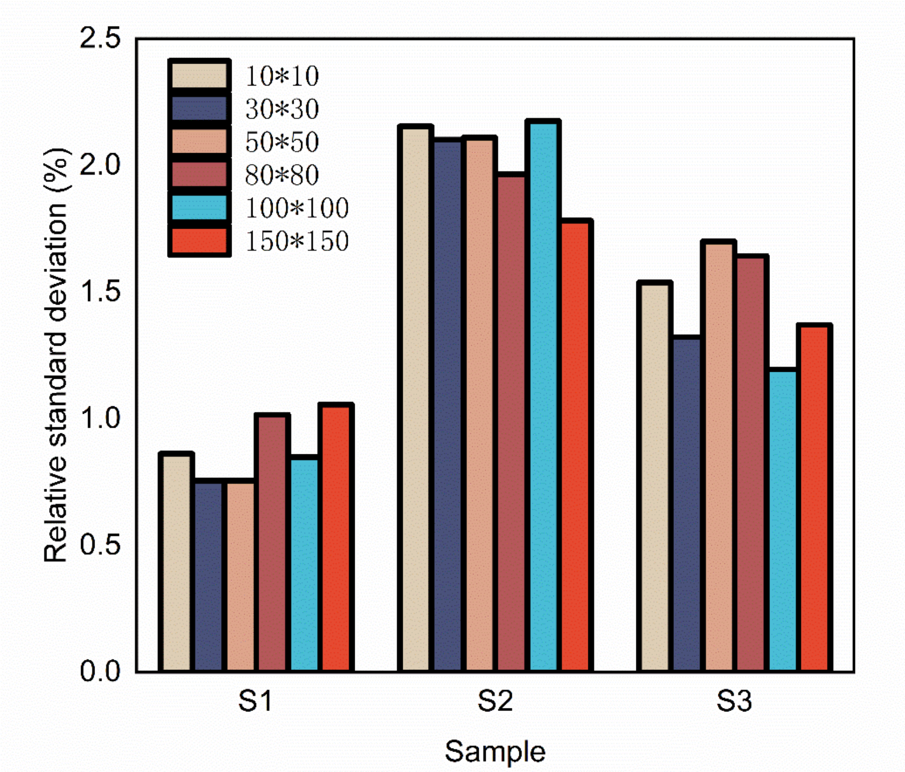

Fig. S21. Optimization of the color picking range in the developed app, including 10*10, 30*30, 50*50, 80*80, 100*100 and 150*150 pixels. The relative standard deviation was calculated by measuring 60 samples with our app (analyte concentrations covering the whole linear range). It is calculated as the standard deviation over the average value.

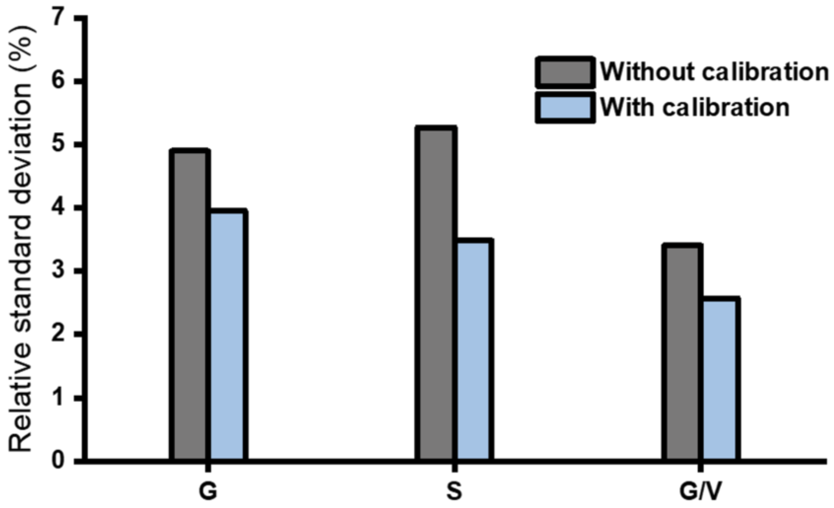

Fig. S22. Algorithmic equalization, where optimization is measured by the relative standard deviation, with no calibration (gray bars) and with calibration (blue bars).

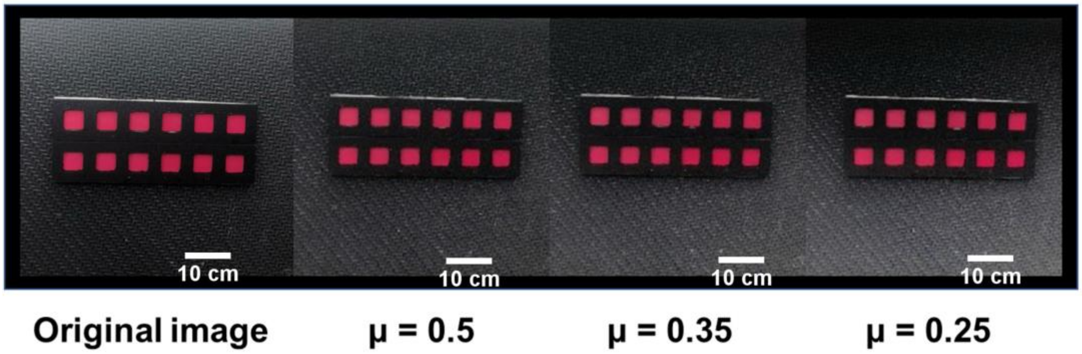

Fig. S23. Visualization of algorithm effect under different μ values.

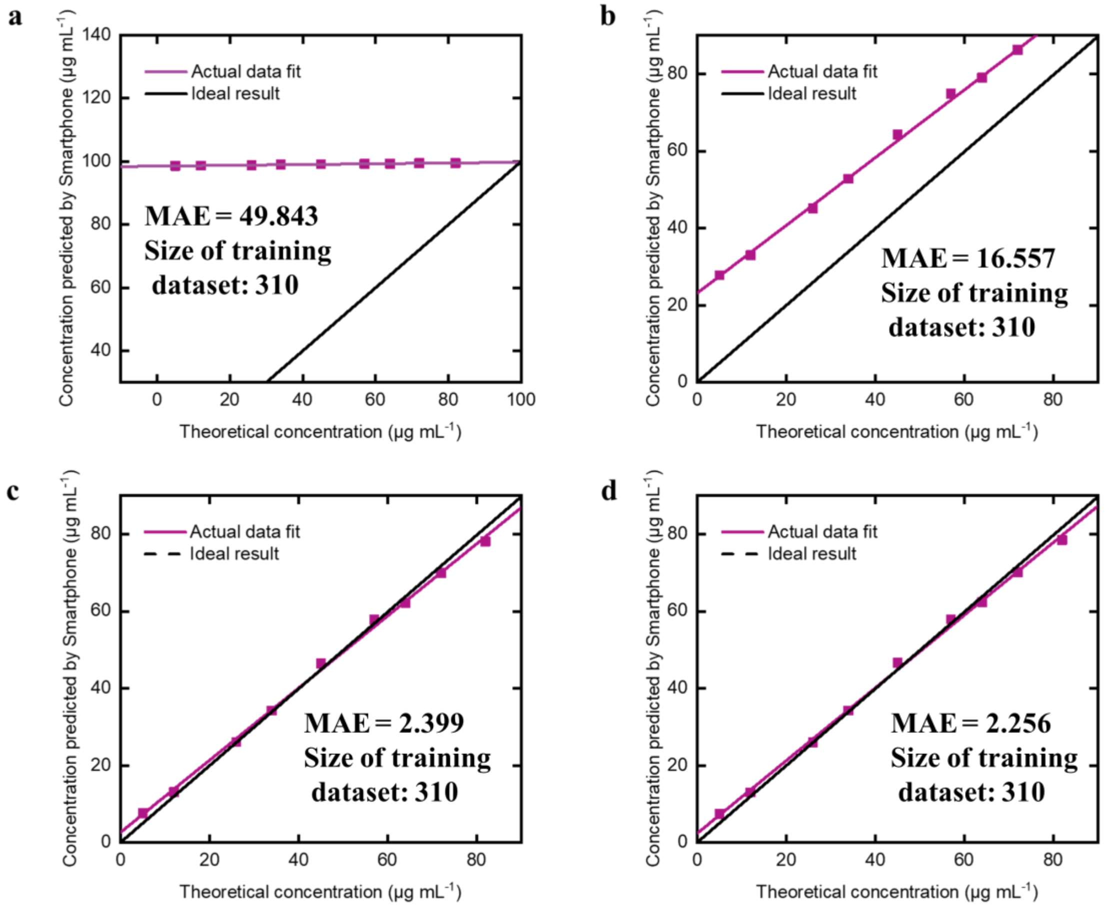

Fig. S24. Evaluation of the accuracy of our machine learning model by training with random subsamples (with different environmental conditions).

Table S1. Average value (V) of samples taken in different backgrounds.

**Background**	**V (%)**
Black	96.72 (±0.16, n=6)
Red	68.76 (±0.6, n=6)
Green	66.41 (±2.2, n=6)
White	61.03 (±0.59, n=6)

Table S2. Skin phototype mapping with subjects. Gray labels highlight the misdiagnosed patients by pulse oximeter.

ID	MI	EHSCS Quantile	L*	Fitzpatrick Type	Von Luschan's Chromatic Scale
1	29.14	2	65.43	IV-V	16-28
2	28.05	2	66.79	IV-V	16-28
3	25.78	2	69.43	IV-V	16-28
4	26.39	2	68.91	IV-V	16-28
5	28.90	2	67.54	IV-V	16-28
6	32.86	2	62.99	V-VI	22-36
7	31.37	2	63.82	V-VI	22-36
8	28.85	2	67.07	IV-V	16-28
9	32.60	2	62.93	V-VI	22-36
10	31.56	2	63.91	V-VI	22-36
11	29.26	2	65.88	IV-V	16-28
12	30.03	2	62.55	V-VI	22-36
13	27.20	2	68.61	IV-V	16-28
14	27.74	2	66.87	IV-V	16-28
15	26.97	2	68.47	IV-V	16-28
16	22.55	1	74.25	II-III	6-15
17	25.68	2	71.75	III-IV	16-28
18	29.27	2	65.23	IV-V	16-28
19	55.13	3	44.11	V-VI	22-36
20	53.12	3	47.20	V-VI	22-36
21	26.52	2	71.94	III-IV	16-28
22	43.76	2	53.21	V-VI	22-36
23	62.44	3	41.90	V-VI	22-36
24	22.45	1	75.85	II-III	6-15
25	22.45	1	73.22	II-III	6-15
26	25.11	2	69.25	III-IV	16-28

Table S3. Skin color data (inner upper arm) obtained by spectrophotometer (standard), smartphone imaging and correction.

**ID**	**L* (Standard)**	**L* (Smartphone imaging)**	**L* (Correction)**
1	65.43	55.17	64.70
2	66.79	56.50	67.99
3	69.43	55.33	65.25
4	68.91	64.83	70.18
5	67.54	61.33	66.30
6	62.99	62.17	62.17
7	63.82	59.67	62.67
8	67.07	53.50	67.69
9	62.93	57.83	65.74
10	63.91	57.50	63.19
11	65.88	57.17	67.79
12	62.55	47.33	56.42
13	68.61	59.00	68.63
14	66.87	55.50	69.14
15	68.47	59.00	69.90
16	74.25	63.00	75.18
17	71.75	58.00	72.57
18	65.23	55.33	67.81
19	44.11	47.50	47.76
20	47.20	51.83	52.91
21	71.94	59.67	70.66
22	53.21	52.17	52.17
23	41.90	41.17	41.17
24	75.85	61.33	72.97
25	73.22	67.00	74.52
26	69.25	67.17	66.64

Table S4. Skin color data (surrounding lip skin) obtained by spectrophotometer (standard), smartphone imaging and correction.

ID	L* (Standard)	L* (Smartphone imaging)	L* (Correction)
1	65.43	41.67	58.74
2	66.79	65.67	65.67
3	69.43	61.67	75.49
4	68.91	78.00	67.45
5	67.54	61.00	70.87
6	62.99	46.33	61.88
7	63.82	66.33	73.25
8	67.07	55.67	65.54
9	62.93	49.00	62.17
10	63.91	64.67	64.67
11	65.88	50.33	60.55
12	62.55	59.00	66.42
13	68.61	68.00	68.00
14	66.87	49.33	67.17
15	68.47	53.67	69.44
16	74.25	48.00	66.20
17	71.75	63.33	70.90
18	65.23	58.67	72.48
19	44.11	37.00	49.10
20	47.20	34.00	49.99
21	71.94	61.67	73.64
22	53.21	29.00	45.31
23	41.90	32.00	44.34
24	75.85	52.33	64.60
25	73.22	43.00	58.42
26	69.25	54.33	73.34

Table S5. RMSE of SpO_2 measured by different method within subjects of different skin phototypes.

**Skin phototypes**		**RMSE with gold standard (%)**		
Fitzpatrick Type	Von Luschan's Chromatic Scale	Pulse oximeter measurement without skin tone correction	Pulse oximeter measurement with skin tone correction	Our app measurement
II-III	6-15	2.17	0.77	0.3
III-IV	16-28	2.31	0.93	0.58
IV-V	16-28	2.82	1.3	0.69
V-VI	22-36	3.16	1.62	0.82

Table S6. RMSE of SpO_2 measured by different method within misdiagnosed patients (See gray labels in Table S3) of different skin phototypes.

Skin phototypes		RMSE with gold standard (%)		
Fitzpatrick Type	Von Luschan's Chromatic Scale	Pulse oximeter measurement without skin tone correction	Pulse oximeter measurement with skin tone correction	Our app measurement
II-III	6-15	2.78	1.11	0.35
III-IV	16-28	3.24	1.28	0.78
IV-V	16-28	3.73	1.67	0.9
V-VI	22-36	5.17	2.74	1.3

Table S7. Comparison of different intelligent oximetry approaches.

Source	Accuracy (MAE <1.5%)	Gold-standard (brand)	Samples	study protocol	Cost (<$10)	Skin tone and melanin-based correction	Specific condition required
Hoffman, J.S. et al.[5]	X	Pulse oximeter (Masimo Radical-7)	6 healthy test subjects	gas mixture to induce hypoxemia	✓	X	Kept at a constant level
Mohammad et al.[6]	✓	Pulse oximeter (model: PO30)	25 healthy subjects	n/a	X	X	n/a
Kaviya et al.[7]	X	Pulse oximeter (Dr trust)	3 healthy individuals	n/a	✓	X	n/a
Wei Lu et al.[8]	✓	Pulse oximeter (DASH 3000)	4 subjects (different skin photo-type)	breath hold	X	X	n/a
Lamonaca et al.[9]	X	Pulse oximeter (CMS50D+)	20 Healthy volunteers, 6 patients	n/a	✓	X	Covered by a black drape
Phelps et al.[10]	X	Pulse oximeter (Masimo RAD 87	3 test subjects	n/a	X	X	Customized device
Holz et al.[11]	\	Oximeter (Nonin Onyx II)	n/a	n/a	✓	X	Shielded
Ding et al.[12]	X	Medical pulse oximeter (Nellcor PM10N)	39 participants without pulmonary or heart disease	breath hold	✓	X	A controlled room
Raposo et al.[13]	X	Oximeter (CMS50D+)	10 healthy subjects	breath hold	✓	X	Black boundary
Kateu et al.[14] (SmartPhOx)	✓	CMS50E pulse oximeter	37 healthy subjects	breath hold	✓	X	Evaluated
Our method	✓	Blood-gas tests (RADIOMETER, ABL9)	47 subjects including clinical cardiovascular patients and health subjects	none	✓	✓	none

Table S8. Camera settings. When automatic adjustment was performed, we found that varying ambient illuminance did not have a significant effect on the precision of the results (0.01 < RE < 0.16), Supplementary Figure 4b. As expected, the parameters, ISO and shutter time, adaptively changed with illuminance and overexposure occurred in this mode with a low probability. However, if the camera setting was fixed (ISO: 320, shutter speed: 1/60 s, aperture: f/1.8), the relative error (RE) ranged from 0.01 and 0.21 while illuminance was less than 2000 Lux, but it increased from 0.04 to 1.16 when illuminance > 2000 Lux (Supplementary Figure 4c, b). When overexposure appeared, this resulted in image distortion of lower concentration samples and signals of true color were lost (Supplementary Figure 13c).

ISO	Shutter time	Illuminance (Lux)
64	1/50	4490
100	1/50	2710
160	1/50	1340
250	1/33	662
320	1/33	386

Table S9. Reagents

Reagent	CAS	Source
Emodin	518-82-1	NIFDC (China)
Glucose oxidase	9001-37-0	Macklin Inc. (China)
Horseradish peroxidase	9003-99-0	
TMB	219322-86-8	
Glucose	5996-10-1	Sinopharm Chemical Reagent Co., ltd. (China)
NaOH	1310-73-2	
Ferric trichloride	7705-08-0	
Copper sulfate	7758-98-7	
Sulfuric acid	7664-93-9	
NH_4OH	1336-21-6	Aladdin® (China)
Acetic acid	64-19-7	
Ammonium acetate	631-61-8	

Movie S1.
Demonstration of the App measuring a patient.

Movie S2
Demonstration of the analysis of standards of different colors utilizing the app.

Movie S3.
Demonstration of loading samples and measuring them using the app.

SI References

1. Collings, S.; Thompson, O.; Hirst, E.; Goossens, L.; George, A.; Weinkove, R., Non-Invasive Detection of Anaemia Using Digital Photographs of the Conjunctiva. *PLoS One* **2016,** *11* (4), e0153286.
2. Schneider, C. A.; Rasband, W. S.; Eliceiri, K. W., NIH Image to ImageJ: 25 years of image analysis. *Nat Methods* **2012,** *9* (7), 671-5.
3. Wang, T. T.; Lio, C. K.; Huang, H.; Wang, R. Y.; Zhou, H.; Luo, P.; Qing, L. S., A feasible image-based colorimetric assay using a smartphone RGB camera for point-of-care monitoring of diabetes. *Talanta* **2020,** *206*, 120211.
4. Cherri, A. K.; Karim, M. A., Optical symbolic substitution: edge detection using Prewitt, Sobel, and Roberts operators. *Applied optics* **1989,** *28* (21), 4644-8.
5. Hoffman, J. S.; Viswanath, V. K.; Tian, C.; Ding, X.; Thompson, M. J.; Larson, E. C.; Patel, S. N.; Wang, E. J., Smartphone camera oximetry in an induced hypoxemia study. *NPJ Digit Med* **2022,** *5* (1), 146.
6. Hoseinzadeh, M. S.; Ekhlasi, A. In *A Wireless Body Temperature and Oxygen Saturation Monitoring system based on Android Smartphones*, 2022 Sixth International Conference on Smart Cities, Internet of Things and Applications (SCIoT), 14-15 Sept. 2022; 2022; pp 1-5.
7. Kaviya, D. A. S.; Jeeva, J. B., Analysis of i-PPG signals acquired using smartphones for the calculation of pulse transit time and oxygen saturation. In *2023 10th International Conference on Signal Processing and Integrated Networks (SPIN)*, 2023; pp 148-153.
8. Lu, W.; Bai, W.; Zhang, H.; Xu, C.; Chiarelli, A. M.; Vázquez-Guardado, A.; Xie, Z.; Shen, H.; Nandoliya, K.; Zhao, H.; Lee, K.; Wu, Y.; Franklin, D.; Avila, R.; Xu, S.; Rwei, A.; Han, M.; Kwon, K.; Deng, Y.; Yu, X.; Thorp, E. B.; Feng, X.; Huang, Y.; Forbess, J.; Ge, Z.-D.; Rogers, J. A., Wireless, implantable catheter-type oximeter designed for cardiac oxygen saturation. *Science Advances 7* (7), eabe0579.
9. Lamonaca, F.; Carnì, D. L.; Grimaldi, D.; Nastro, A.; Riccio, M.; Spagnolo, V. In *Blood oxygen saturation measurement by smartphone camera*, 2015 IEEE International Symposium on Medical Measurements and Applications (MeMeA) Proceedings, 7-9 May 2015; 2015; pp 359-364.
10. Phelps, T.; Jiang, H.; Hall, D. A. In *Development of a smartphone-based pulse oximeter with adaptive SNR/power balancing*, 2017 39th Annual International Conference of the IEEE Engineering in Medicine and Biology Society (EMBC), 11-15 July 2017; 2017; pp 3297-3300.
11. Holz, C.; Ofek, E. In *Doubling the Signal Quality of Smartphone Camera Pulse Oximetry Using the Display Screen as a Controllable Selective Light Source*, 2018 40th Annual International Conference of the IEEE Engineering in Medicine and Biology Society (EMBC), 18-21 July 2018; 2018; pp 1-4.
12. Ding, X.; Nassehi, D.; Larson, E. C., Measuring Oxygen Saturation With Smartphone Cameras Using Convolutional Neural Networks. *IEEE Journal of Biomedical and Health Informatics* **2019,** *23* (6), 2603-2610.
13. Raposo, A.; Silva, R.; Rosário, L. B.; Sanches, J.; da Silva, H. P., Smartphone Pulse Oximetry Using Two-tone Camera-based Photoplethysmography. In *2023 IEEE 7th Portuguese Meeting on Bioengineering (ENBENG)*, 2023; pp 40-43.

14. Kateu, F.; Jakllari, G.; Chaput, E., SmartPhOx: Smartphone-Based Pulse Oximetry Using a Meta-Region Of Interest. In *2022 IEEE International Conference on Pervasive Computing and Communications (PerCom)*, 2022; pp 130-140.
